# Elevated Aβ aggregates in feces from Alzheimer’s disease patients: a proof-of-concept study

**DOI:** 10.1186/s13195-024-01597-3

**Published:** 2024-10-14

**Authors:** Marlene Pils, Alexandra Dybala, Anja Schaffrath, Fabian Rehn, Janine Kutzsche, Lara Blömeke, Markus Tusche, Pelin Özdüzenciler, Tuyen Bujnicki, Victoria Kraemer-Schulien, Hannes Gramespacher, Maximilian H.T. Schmieschek, Michael T. Barbe, Oezguer A. Onur, Gereon R. Fink, Gültekin Tamgüney, Oliver Bannach, Dieter Willbold

**Affiliations:** 1 Institute of Biological Information Processing, Structural Biochemistry (IBI-7), Forschungszentrum Jülich, 52428 Jülich, Germany; 2 attyloid GmbH, 40225 Düsseldorf, Germany; 3 Institut für Physikalische Biologie, Heinrich-Heine-Universität Düsseldorf, 40225 Düsseldorf, Germany; 4 Department of Neurology, Faculty of Medicine and University Hospital Cologne, University of Cologne, 50923 Köln, Germany; 5 Cognitive Neuroscience, Institute of Neuroscience and Medicine (INM-3), Jülich Research Centre, 52428 Jülich, Germany

**Keywords:** Amyloidosis, Aβ oligomer quantitation, sFIDA, Brain-gut-microbiota axis, Leaky gut, Fecal/stool samples, Clearance

## Abstract

**Background:**

Misfolding and aggregation of amyloid β (Aβ), along with neurofibrillary tangles consisting of aggregated Tau species, are pathological hallmarks of Alzheimer’s disease (AD) onset and progression. In this study, we hypothesized the clearance of Aβ aggregates from the brain and body into the gut.

**Methods:**

To investigate this, we used surface-based fluorescence intensity distribution analysis (sFIDA) to determine the Aβ aggregate concentrations in feces from 26 AD patients and 31 healthy controls (HC).

**Results:**

Aβ aggregates were detectable in human feces and their concentrations were elevated in AD patients compared to HC (specificity 90.3%, sensitivity 53.8%).

**Conclusion:**

Thus, fecal Aβ aggregates constitute a non-invasive biomarker candidate for diagnosing AD. Whether digestion-resistant Aβ aggregates in feces are secreted via the liver and bile or directly from the enteric neuronal system remains to be elucidated.

**Supplementary Information:**

The online version contains supplementary material available at 10.1186/s13195-024-01597-3.

## Background

Alzheimer’s disease (AD) is the most prevalent age-related cause of dementia, characterized by neurodegenerative processes ultimately leading to neuronal loss in the hippocampus and cerebral cortex. Due to neurodegeneration, a progressive decline of cognitive functions, especially learning and memory, is observed [1]. AD is neuropathologically characterized by the progressive accumulation of extracellular senile plaques composed of fibrillar amyloid β (Aβ) peptides and of intracellular neurofibrillary tangles composed of tau proteins [2, 3]. Furthermore, recent evidence indicates that smaller soluble Aβ protein and tau aggregates like oligomers cause and promote pathological processes due to neurotoxicity [3, 4].

There is a growing body of experimental and clinical data confirming a link between the gut, gut microbiota, and neurodegeneration in various neurodegenerative diseases such as AD. In particular, the gut microbiota as a source of a large amount of bacterial amyloids, lipopolysaccharides, short fatty acids and secondary bile acids may promote system inflammation and increase the permeability of physiological barriers. There is even evidence that the gut microbiota is altered both taxonomically and functionally in AD, even before the onset of amyloid pathology in the CNS [5–8]. However, knowledge about changes of intestinal/fecal Aβ is limited. Considering additional factors like disturbances along the brain-gut-microbiota axis [5, 6, 9–11], liver-mediated Aβ clearance and elimination by bile [12–14], and the consequences of a disturbed blood-brain barrier and a permeable intestinal barrier [5, 10, 15, 16], the presence of Aβ aggregates in feces can be assumed. Initial studies have confirmed an association between AD and increased intestinal or fecal Aβ concentrations, irrespectively of conformational structure [17–20]. Protein aggregation occurs in various neurodegenerative disorders, and it often precedes the appearance of clinical symptoms for several years or even decades [21]. Therefore, we hypothesized that clearance mechanisms must exist that reduce the aggregate load in the brain and body by disposing Aβ aggregates via gut. Consequently, fecal Aβ aggregates may also serve as a biomarker candidate for non-invasive AD diagnosis.

**Fig. 1:**
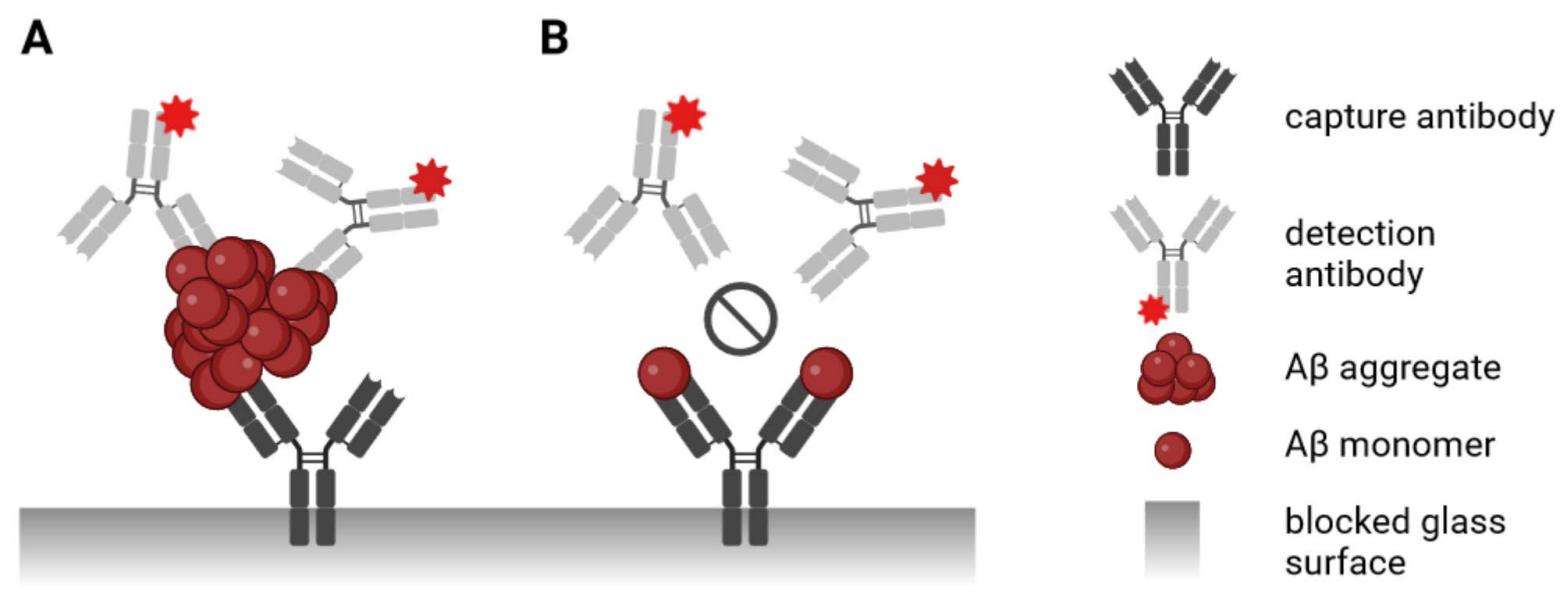
Scheme of sFIDA principle. In sFIDA, capture antibodies directed against a linear epitope on Aβ (Nab228, directed against epitope amino acids 1 − 11) are immobilized on a glass surface, and unoccupied surface area is blocked with bovine serum albumin to reduce unspecific binding events. During sample incubation, monomeric and aggregated Aβ species are bound to the capture antibody. (**A**) Because sFIDA uses the same or overlapping epitopes for capture and detection, only Aβ aggregates are subsequently detected with fluorescence-labeled antibodies IC16-CF633, which is overlapping with the epitope of the capture antibody (directed against epitope amino acids 2 − 8). (**B**) For monomeric Aβ, this epitope is already masked by the capture antibody and cannot be bound by the detection antibody. Afterward, the assay surface is imaged by fluorescence microscopy, and pixels above a defined cutoff threshold are counted by image-data analysis (called pixel count). Finally, pixel-based readouts are calibrated into molar particle concentrations using silica nanoparticles (SiNaPs) coated with Aβ as calibration standards. Created with BioRender.com

We previously developed surface-based fluorescence intensity distribution analysis (sFIDA), a platform technology for quantitating single protein aggregates [22]. While the biochemical setup of the sFIDA assay is similar to a sandwich ELISA (Fig. 1), the readout is microscopy-based featuring sub-femtomolar sensitivity [22, 23]. To avoid monomer interference, sFIDA uses capture and detection antibodies directed against the same or overlapping epitopes. After probing, the glass surface is imaged by total internal reflection fluorescence microscopy (TIRFM), illuminating fluorescence-labeled detection antibodies bound to the captured aggregates. Individual particles are counted by image-data analysis of pixels with fluorescence intensities above background noise. Our previous work established the technical concept of sFIDA [23–25] and demonstrated that sFIDA is useful for diagnosing neurodegenerative diseases [26–28] and drug development [29].

This study did not intend to investigate the gut microbiome, but to investigate, whether Aβ aggregates are found in human feces and whether their levels are different in AD diseased donors versus healthy controls (HC). Therefore, we developed and analytically validated the sFIDA assay to detect and quantify Aβ aggregates in human fecal samples. We also assessed its applicability as an explorative biomarker for non-invasive AD diagnosis in a small proof-of-concept study including samples from 26 AD patients and 31 HC.

## Methods

### Human fecal samples

**Fig. 2:**
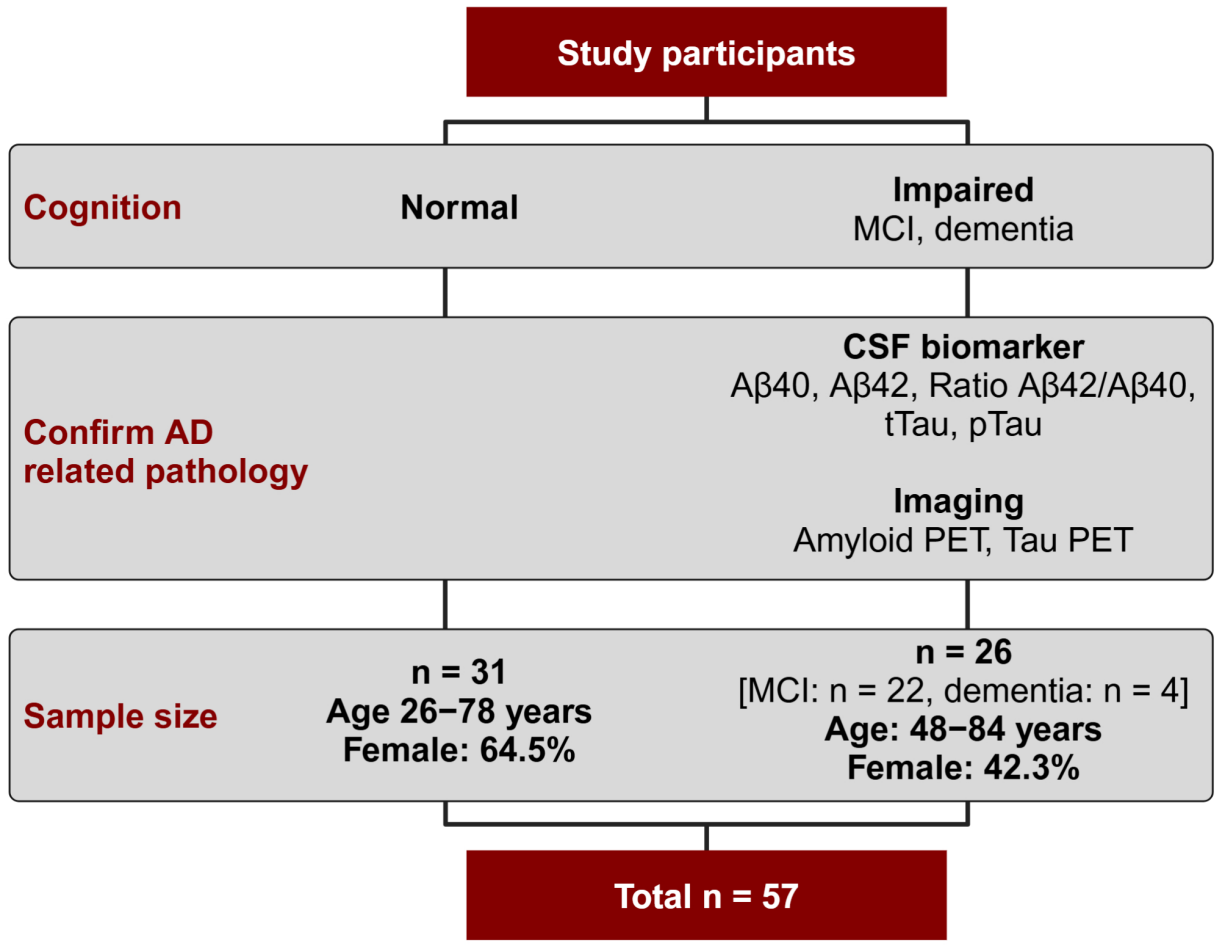
Schematic illustration of the sample collection process. Study participants were divided into two groups (normal cognition, impaired cognition) based in their clinical symptoms. CSF analyses or imaging were performed for the participants with impaired cognition, to confirm AD related pathology. In total, 31 HC and 26 participants with impaired cognition due to amyloid pathology were included into this study. Created with BioRender.com

Fecal samples of study participants were collected between October 2019 and June 2022. The Ethics Commission of the Faculty of Medicine of the University of Cologne approved patient recruitment (19-1644). Informed consent was obtained from each participant. A schematic illustration of the sample collection process is shown in Fig. 2.

HC had no known neurological disease, were not subjectively cognitively impaired, and showed complete functional abilities in daily living (ADL). The participants showing cognitive decline were diagnosed by an interdisciplinary team of clinicians with extensive experience in dementia care and research. The neuropsychological classification of the patients involved the following test procedures: DemTect [30], used as a screening tool to identify patients with mild cognitive impairment (MCI) and early-stage dementia; the Beck Depression Inventory II (BDI-II) [31] to evaluate the presence and severity of depression; the Memory Assessment Clinic-Questionnaire (MAC-Q) [32], a brief questionnaire for assessing age-related memory decline; and the Functional Activities Questionnaire (FAQ) to evaluate the ability to perform ADL [33]. The results of the neuropsychological tests for each cognitively impaired participant are listed in Supplementary Table S1. Cognitive impaired participants had to show the typical clinical syndrome according to the National Institute on Aging and Alzheimer’s Association (NIA-AA) guidelines [34]. These guidelines specify that patients should have a subtle onset and slow progression of cognitive impairment (either self-reported or reported by a third party) and memory deficits greater than 1.5 standard deviations below the average in any neuropsychological testing results, adjusted for age, sex, and education. In this study, the cognitively impaired participants with preserved functional ADL, as assessed by the FAQ [33] or, in the absence of the FAQ, based on the medical history and interview of the caregiver by an experienced clinician, were classified as having MCI. If there were indications of impaired ADL, cognitively impaired participants were classified as having dementia. In order to check whether MCI and dementia are due to AD, biomarker-based diagnostics were carried out.

As described in the research framework of the NIA-AA, AD can be uniformly defined biologically, capable of identifying early pathological changes and biomarker interactions associated with the disease. Therefore, individuals can now be placed in the AD continuum as soon as pathological Aβ aggregation occurs, regardless of their cognitive status [1]. Reduced levels of Aβ42, a reduced ratio between Aβ42 and Aβ40 as well as the image-based detection of senile plaques using amyloid PET serve as evidence for the presence of Aβ aggregation. In the present study, positive amyloid PET (by visual read) and/or CSF levels of Aβ42 less than 630 pg/mL (ELISA kit of Euroimmun AG, Lübeck, Germany, product ID EQ 6511-9601-L, grey zone 570 − 630 pg/mL) and/or a CSF ration between Aβ42 and Aβ40 less than 0.095 (ELISA kit of Euroimmun AG, product ID EQ 6521-9601-L) confirmed amyloid positivity of cognitively impaired participants and classified them as clinical diagnosed AD patients. To confirm tauopathy, we determined levels of pTau (Fujirebio Europe N.V., Gent, Belgium, product ID 81574, cut-off > 61 pg/mL) and tTau (Euroimmun AG, product ID EQ 6531-9601-L, cut-off > 452 pg/mL, grey zone 290 − 452 pg/mL) in CSF as well as tau positivity via PET scan (by visual read).

Based on amyloid positivity, 26 patients (48 − 84 years of age at sample collection, 42.3% females) were enrolled as clinically defined Alzheimer’s disease patients with varying degrees of cognitive impairment (*n* = 22 patients with mild cognitive impairment (MCI), *n* = 4 patients with dementia). In total, 31 samples from HC were collected (26 − 78 years of age at sample collection, 64.5% females) and were transferred to sFIDA laboratory without disclosing the names of the donors.

#### Sample collection

Fecal samples were collected using polypropylene sample tubes with a screwcap-integrated spoon (megro, Wesel, Germany) and a paper-based collection aid (Med Auxil analysis aids, Seesen, Germany) to avoid contamination. In order not to endanger sample stability, samples were stored and transported on ice. After receiving the fecal samples, samples were classified according to their consistency and shape using the Bristol scale [35] and were aliquoted into polypropylene protein low-binding tubes (Sarstedt, Nümbrecht, Germany). Samples were stored at − 80 °C until further use.

#### Homogenization of fecal samples for sFIDA analysis

For sample homogenization, we have developed a sample homogenization buffer and established a suitable homogenization method. A detailed description can be found in the Supplementary method section and Supplementary Figure S1. Fecal homogenates were stored at − 80 °C until further use.

### sFIDA Assay

#### Synthesis of Aβ1 − 15 coated silica nanoparticles

For assay calibration, we have previously introduced silica nanoparticle (SiNaP) standards coated with multiple Aβ-derived epitopes to mimic Aβ aggregates [24]. To this end, bare SiNaPs were synthesized via the Stöber process, functionalized, and activated as described previously by Blömeke et al. [26]. Briefly, synthesized SiNaPs were silanized with 3aminopropyl(triethoxysilane) (APTES, Sigma-Aldrich, St. Louis, MO, USA) to functionalize the surface with primary amino groups. Afterward, crosslinking of Aβ1 − 15 peptides to aminated SiNaPs surface was enabled using maleimido hexanoic acid (MIHA, abcr GmbH, Karlsruhe, Germany), preactivated with 1-ethyl-3-(3-dimethylaminopropyl)carbodiimide (EDC, Sigma-Aldrich) and N-hydroxysuccinimid (NHS, Sigma-Aldrich). Using C-terminal functionalized Aβ1 − 15 peptides with cysteamine, crosslinking between Aβ and maleimide groups was enabled. Finally, molar concentration of Aβ-coated SiNaPs was calculated based on silicon concentration determined by inductively coupled plasma-mass spectrometry, density of SiNaPs and size and shape of the particles, as determined by transmission electron microscopy.

#### Aβ1 − 42 oligomer-based IQC sample

We have previously introduced synthetic Aβ1 − 42 oligomers as internal quality control (IQC) sample [25]. To this end, 5 µg of monomeric Aβ1 − 42 (Bachem, Bubendorf, Switzerland) was dissolved in 1,1,1,3,3,3-hexafluoro-2-propanol (HFIP, Sigma-Aldrich) and evaporated. Afterward, the Aβ pellet was resolved with 5 µL dimethyl sulfoxide (DMSO, Sigma-Aldrich), agitated for 10 min at 650 rpm at RT and diluted with 1× PBS containing 0.04% NaN_3_ to a final stock concentration of 10 µM. Following an overnight incubation on a shaker at 650 rpm and RT, the IQC sample was sonicated for 20 min in an ultrasonic bath before dilution and use in sFIDA assay.

#### Labeling of antibody

For detection of captured Aβ aggregates, fluorescence-labeled anti-Aβ antibody IC16 (directed against epitope amino acid 2 − 8, kindly provided by Carsten Korth, Universitätsklinikum Düsseldorf, Germany) [36] was applied on the assay surface. To this end, the antibody was labeled according to the manufacturer’s protocol using CF633 dye (Biotium, Freemont, CA, USA). In carbonate buffer, the succinimidyl ester groups of the preactivated dye bind covalently to the amines of the IC16 antibody. Purification was performed using a polyacrylamide bead suspension (Bio-Gel P-30 Gel, Bio-Rad Laboratories Inc, Hercules, CA, USA), and afterward, concentration and degree of labeling of the probe were calculated as described in manufacturer’s protocol. Finally, the detection probe was stored at 4 °C, diluted, and centrifuged for 1 h at 4 °C and 100,000× *g* just before usage.

#### Assay protocol

The biochemical principle of sFIDA was reported previously [23, 24, 26]. In this study, 384-well-plates (SensoPlate plus, Greiner Bio-One, Frickenhausen, Germany) were functionalized with 40 µL of Nab228 monoclonal anti-Aβ antibody (Sigma-Aldrich) at 2.5 µg/mL in 0.1 M NaHCO_3_. After overnight incubation at 4 °C, the wells were washed five times with TBST (1× Tris-buffered saline, TBS (Serva, Duisburg, Germany), 0.05% Tween20 (AppliChem, Darmstadt, Germany)) and five times with TBS. Non-coated glass area was blocked with 0.5% bovine serum albumin (BSA, AppliChem) in TBS-ProClin (TBS with 0.03% ProClin (Sigma-Aldrich)) for 1.5 h at RT. After washing five times with TBST and TBS, 20 µL samples were applied in 4-fold determination and incubated for 2 h at RT. For this, Aβ-coated SiNaPs as calibration standard and synthetic Aβ1 − 42 oligomers as IQC were diluted in sample buffer (1× phosphate-buffered saline (PBS, Sigma-Aldrich), 0.05% Tween, 0.095% NaN_3_ (AppliChem) and 0.5% BSA). For fecal samples, an assay-specific 1:5 dilution in sample buffer was performed. After washing five times with TBS, 20 µL of IC16-CF633 (0.625 µg/mL in TBST + 0.1% BSA) were applied and incubated for 1 h at RT. Finally, wells were washed five times with TBS, and buffer was changed against TBS-ProClin.

#### Image data acquisition

Using TIRFM (Leica DMI6000B, Leica microsystems, Wetzlar, Germany), 3.15% of the well surface were imaged at 25 different positions (14-bit grayscale, excitation: 635 nm, emission filter: 705/72 nm, exposure time: 1000 ms, gain: 1000). Imaging of 3.15% of the complete surface area accounts for avoiding edge regions and has been shown to be representative for the well [23, 25, 26]. Each image consists of 1000 × 1000 pixels and represents an area of 113.76 × 113.76 μm.

### Quantification and statistical analysis

#### Analysis of image data

Image data analysis was performed using the in-house developed software sFIDAta [23, 26]. All images containing artifacts or images that were out of focus were excluded from analysis. For the exclusion of background signal, an intensity cutoff was determined at which 0.001% of all pixels remain positive in the blank control (unspiked sample buffer, BC). The number of pixels above the respective cutoff is referred to as pixel count. sFIDAta calculated the mean value, standard deviation, and coefficient of variation (CV%) for each sample based on the four replicates. Statistical analyses were performed using OriginPro (OriginLab Corporation, Northampton, MA, USA) and matlab2019b (The MathWorks, Natick, MA, USA) were used for calculations and graphs.

#### Calibration

To convert pixel counts into femtomolar particle concentration, we used readouts of Aβ-coated SiNaPs to calculate the calibration curve. To this end, only those Aβ-coated SiNaPs concentrations which significantly differed from BC and were within linear range were included in the calculation. A one-sided Mann − Whitney U test with a confidence interval of 5% was performed to investigate significant differences. Linear regression was executed with matlab2019b software, where pixel counts were weighted with 1/readout.

#### Preanalytics

As it was the first time that we analyzed stool samples to quantify Aβ aggregates using sFIDA, we performed several studies to confirm reproducibility of the preanalytical processes, including fecal sample homogenization and sample dilution. We also investigated the effect of different transport conditions as well as the effect of repeated freeze-thaw cycles on sample stability. A detailed description of these preanalytical study designs is listed in the Supplementary Methods.

#### Analytical validation

##### Assay selectivity

To evaluate the selectivity of the sFIDA assay in detecting Aβ-coated SiNaPs (molar particle concentration of 10.26 pM), IQC samples (100 nM, Aβ monomer subunit concentration), and three fecal samples with intermediate to high readouts, we measured the percent signal reduction of capture, autofluorescence, and cross-reactivity control, and compared it to a standard assay setup. In addition, we also performed immunodepletion experiments to determine if the observed pixel counts were specifically attributed to Aβ aggregates and not to interfering fecal matrix components. A detailed description of the experiments is listed in the Supplementary Method section.

##### Influence of Bristol scale

Recent studies have demonstrated that microbiome composition and species richness change during AD progression and might impact cognition [6, 7, 37, 38]. Microbiome composition and species richness also affect feces consistency. Therefore, changes in both water content and pH value are directly reflected in the Bristol scale [35, 39]. The latter may also act as a non-analyte-specific interfering factor [40], altering the quantification of the analyte through dilution or pH-dependent changes in assay kinetics. At sample receipt, all samples were assessed according to their consistency and shape using the Bristol scale. We performed a Mann − Whitney U test and a Spearman correlation analysis to determine whether the Bristol scale influences the fecal Aβ aggregate concentration or whether a cognition-based influence is present.

##### Influence of matrix components

The presence of interfering endogenous substances in fecal samples may falsely alter assay results, either falsely positive or falsely negative [41, 42]. Because disturbances of the brain-gut-microbiota axis, including gut inflammation and increased permeability of the intestinal barrier, may contribute to AD pathology [5], levels of fecal biomarkers indicating pathological processes of the gut, i.e., fecal albumin, hemoglobin, α-1-antitrypsin, calprotectin, IgA, lipids, and bile acid were determined for a set of 15 fecal samples (AD *n* = 7, HC *n* = 8). Biomarker analyses were performed by the Medizinisches Versorgungszentrum Limbach (Heidelberg, Germany). Afterward, Spearman correlation was conducted to investigate possible interfering effects of those biomarkers on fecal Aβ aggregate concentrations. In addition, Mann − Whitney U tests were used to determine whether levels of all seven biomarkers differ in AD patients and HC.

##### Intra- and inter-assay variability

The precision of an analytical method refers to the consistency of the measured values of several replicates of the same sample. This precision can be categorized as intra-assay variability when considering measurements within a single assay, or inter-assay variability when comparing measurements across different assays [43]. In this study, each sample was analyzed in fourfold determination. For each sample, mean and standard deviation were calculated based on the pixel counts of the four replicates. The intra-assay variation is reflected by CV% value, while for synthetic Aβ species such as Aβ-coated SiNaPs and IQC sample values below 20% were accepted. In case of human fecal samples, CV% below 25% were accepted. Normally, inter-assay variance is determined through a comprehensive validation study. However, due to the limited sample volume available in the present study, we were unable to conduct such an extensive validation. Instead, we assessed the comparability of two independent measurements using Spearman correlation analysis, with Spearman’s ρ above 0.9 indicating low inter-assay variability.

##### Calculation of LoD

In order to describe the smallest concentration of Aβ aggregates that can be measured reliably with the sFIDA assay, the limit of detection (LoD) was estimated for each experiment by measuring 24 replicates of BC. First, LoD was calculated according to Armbruster et al. using Eq. 1 [44] and then calibrated into femtomolar concentrations using the determined calibration line.

1 $${\rm{Limit}}\,{\rm{of}}\,{\rm{detection}}\,\left( {{\rm{LoD}}} \right)\,{\rm{ = }}\,{\rm{pixel}}\,{\rm{coun}}{{\rm{t}}_{{\rm{BC}}}}{\rm{ + 2\sigma }}$$

#### Proof-of-concept study

For the proof-of-concept, the whole set of 57 fecal samples was subjected to sFIDA. At the time of measurements, all patient-related data was anonymized and researchers were aware of the clinical data at the time of the sFIDA measurements since HC samples were transferred to the sFIDA laboratory without the names of the donors. Pixel counts were generated and calibrated. Data of fecal samples were subsequently tested for normal distribution using Shapiro − Wilk, Lilliefors, Kolmogorov Smirnov, and Anderson − Darling tests. In the case of non-normally distributed data, non-parametric tests, e.g., the Mann − Whitney U test or Spearman correlation, were conducted for further analyses. A receiver operating characteristic (ROC) analysis was performed to evaluate the effectiveness of fecal Aβ aggregates as diagnostic biomarker to differentiate between AD patients and HC. Using maximized Youden’s index, the optimal combination of sensitivity and specificity and the area under the curve (AUC) were calculated.

## Results

### Homogenization and sample dilution of fecal samples are reproducible

Because fecal samples must be homogenized before they are subjected to sFIDA, we first established a suitable homogenization buffer and protocol. Due to the complex and individually varying composition of feces, we tested the reproducibility of homogenization and sample dilution (1:5 in sample buffer) using normalized pixel counts of three fecal samples (initial low, intermediate, high readout) to calculate percentage reproducibility. Figure S2 demonstrates that the homogenization and sample dilution resulted in a high degree of reproducibility, as most of the normalized pixel counts fell within the predefined tolerance range of ± 25%. Only two of the observed values have exceeded the tolerance range with a slight deviation of ± 0.8% (Figure S2A).

### sFIDA features dilution linearity of Aβ-coated SiNaPs, IQC Samples, and fecal samples

Next, we analyzed dilution linearity by subjecting an Aβ-coated SiNaPs and IQC dilution series to sFIDA analysis, ranging from 0.32 fM − 10 pM (molar particle concentration) and 3.2 pM − 31 nM (total Aβ concentration), respectively. The percent dilution linearity was determined using blank-corrected pixel counts and was accepted within a tolerance range of 80 − 120%. The results showed high dilution linearity for both targets, with an average linearity of 107% for Aβ-coated SiNaPs and 97.6% for IQC samples. Furthermore, for two fecal samples possessing high endogenous Aβ aggregate concentrations, high parallelism of 99.9% and 85.2%, respectively, was determined as depicted in Figure S3.

### Repeated freeze-thaw cycles do not affect stability of homogenized fecal samples

In order not to compromise the sample stability during transport or assay preparation, we first performed a thermostability study. The results indicated that a temperature of 4 °C or lower did not compromise sample stability for up to 18 h, as depicted in Figure S4. In addition, we investigated the influence of freeze/thawing on crude and homogenized fecal samples since repeated freezing and thawing of clinical samples is known to compromise sample stability [43, 45, 46]. To this end, we subjected three fecal samples to repeated freeze-thaw cycles and determined the level of Aβ aggregates. As shown in Figure S5, the stability of the crude fecal samples was indeed affected by multiple thawing and freezing but remained intact in the homogenized fecal samples.

### sFIDA is selective for aggregated Aβ species

**Fig. 3:**
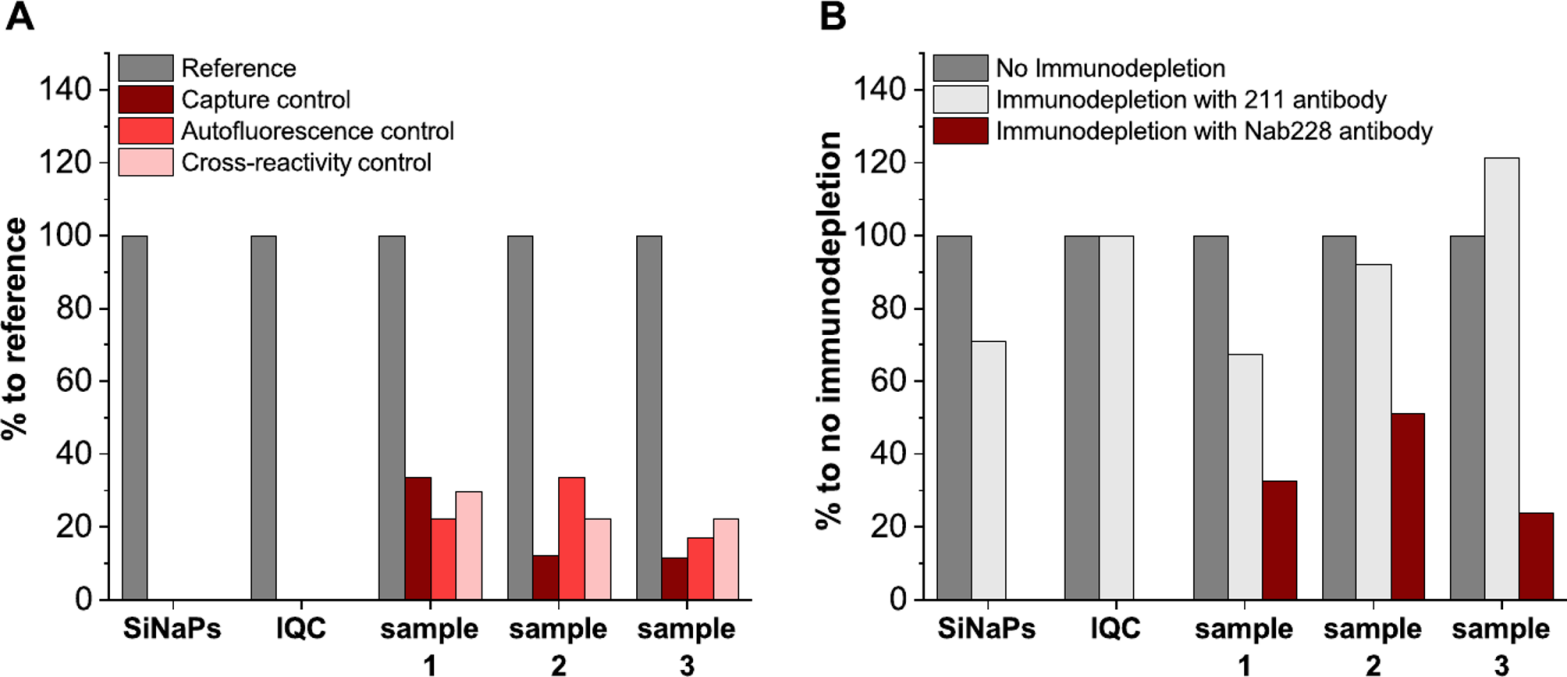
Percent signal reduction of different assay controls for the assessment of assay selectivity. **A**) Aβ-coated SiNaPs, IQC, and Aβ aggregates in three fecal samples were analyzed by sFIDA. Based on the observed pixel counts, the percent signal reduction of each assay control (no capture antibody: capture control, dark red; no detection probe: autofluorescence control, light red; detection probe against α-synuclein: cross-reactivity control, rose) in comparison to reference (standard assay setup) were calculated. (**B**) All samples were subjected to immunodepletion using magnetic beads linked to Aβ-specific antibody (Nab228) and control beads linked to α-synuclein-specific antibody (211). Based on the observed pixel counts of non-depleted (dark gray), 211-depleted (light gray), and Nab228-depleted (red) samples, percent signal reduction was calculated

To evaluate the selectivity of the sFIDA assay, we measured the percent signal reduction of capture, autofluorescence, and cross-reactivity control, and compared it to a standard assay setup (Fig. 3A). In this analysis, the sFIDA assay showed a selectivity of about 100% for both Aβ-coated SiNaPs and IQC samples. Thus, unspecific interference with the used blocking agent, autofluorescent components, and cross-reactivity can be excluded for both targets. In contrast, a selectivity of about 77% was determined for fecal Aβ aggregates. Both capture and cross-reactivity control showed about 19.2% and 24.8% remaining pixel counts, respectively. However, since the absence of the detection probe from the fecal samples also led to a comparable result (24.3% of remaining pixels), higher background intensities of the matrix and no interference with assay surface or cross-reactivity with α-synuclein directed antibodies can be assumed. Despite this increased background noise, the signal reduction down to 25% remaining pixels in the fecal samples was still sufficient.

### Assay readouts are specifically attributed to Aβ

Immunodepletion was performed to demonstrate that the observed pixel counts by sFIDA can be specifically attributed to Aβ aggregates. Following Aβ immunodepletion, supernatants were subjected to sFIDA analysis, and depletion effectivity was calculated by the percent signal reduction compared to non-depleted sample (reference sample). Nab228-depleted Aβ-coated SiNaPs and IQC showed depletion-dependent signal reduction close to 100% (Fig. 3B), whereas, for Nab228-depleted fecal samples, a mean signal reduction of 72.6% was determined. As a control, α-synuclein immunodepletion with 211-coated magnetic beads was performed, resulting in only negligible signal reduction of IQC (0%) and fecal samples (6.5%). However, for Aβ-coated SiNaPs a signal reduction of 28.9% was observed, suggesting some non-specific adherence of Aβ-coated SiNaPs to bead surface since cross-reactivity between 211 and Aβ-coated SiNaPs was previously excluded (Fig. 3A). To qualitatively confirm the presence of Aβ in human fecal samples, we detected Aβ species in two fecal samples (HC and AD) using a commercial ELISA kit (Figure S6). However, differentiation of fecal Aβ levels between the two subjects was only achievable after complex and sample-consuming pretreatment including homogenization, immunoprecipitation, and spiking. In the spike and recovery experiments, the percentage recovery of monomeric 100 pg/mL Aβ1 − 42 spiked in human fecal samples was also increased by eliminating the sample matrix using immunoprecipitation. However, the mean percentage recoveries of the ELISA were only 8.8% (homogenized samples: HC = 3.5%, AD = 14.1%) for homogenized samples and 13.1% (HC = 10.0%, AD = 16.2%) for precipitated samples, indicating substantial assay interference of matrix components that cannot be removed by immunoprecipitation. It is also important to note that the commercial ELISA used in this study was not validated for the analysis of Aβ in fecal samples, which also might explain the rather low recovery rate. In contrast, the sFIDA assay shown here was optimized for the use of stool as sample matrix (cf. homogenization procedure, homogenization buffer and assay-specific sample dilution), so that even at a lower concentration of aggregated Aβ1 − 42 (45 pg/mL spiked in stool sample) a mean percentage recovery of 82.3% was obtained.

### Independent measurements yield comparable results

**Fig. 4:**
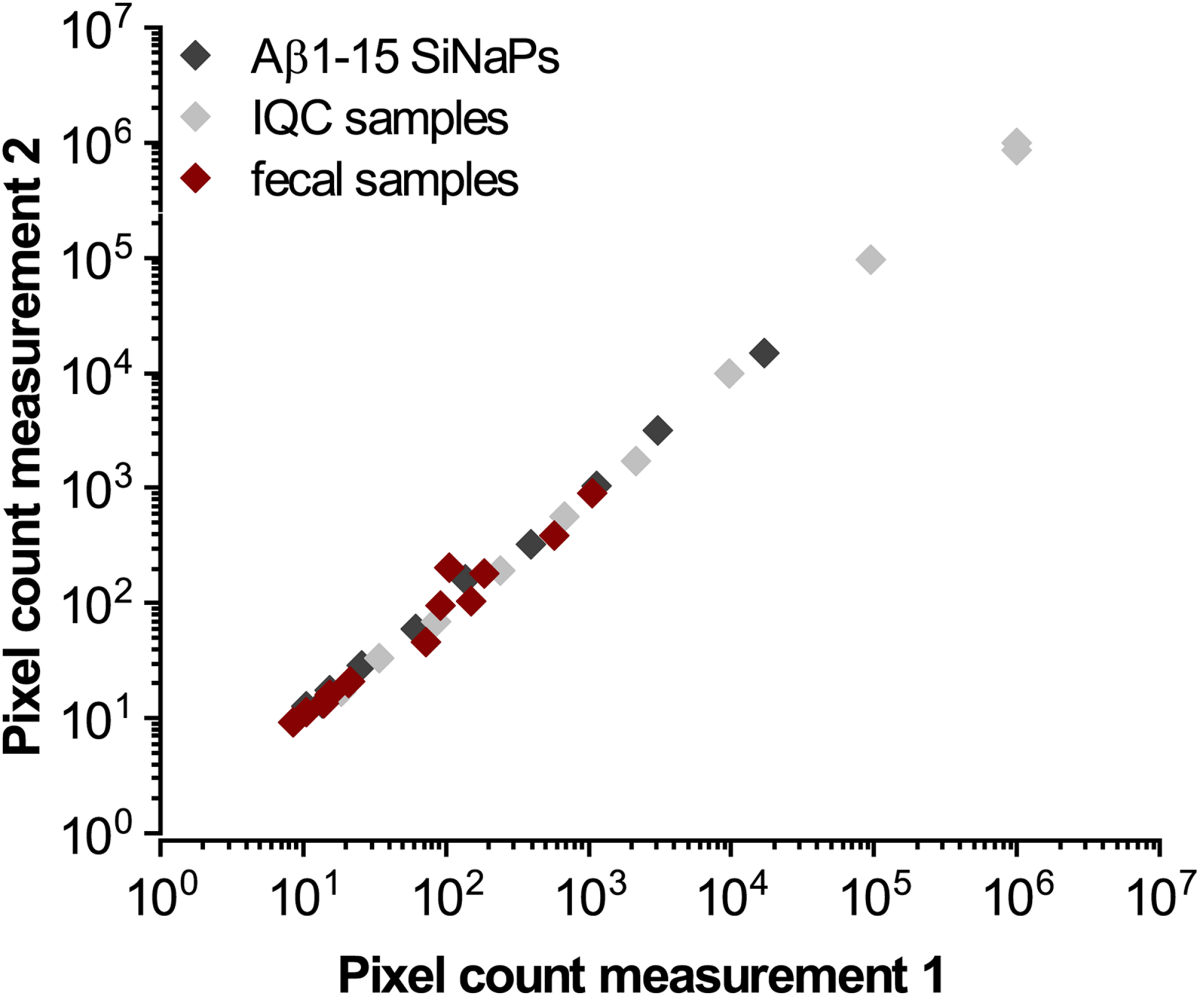
Independent measurements of aggregated Aβ yield high comparability. For Aβ-coated SiNaPs (dark grey), IQC sample (light gray), and 13 fecal samples (red), pixel counts of the second measurement were plotted against pixel counts of the first measurement. Because the second measurement was carried out months later, there were minor changes in used reagent lots, e.g., manufacturing date of washing buffers, and the used homogenized fecal samples were subjected to an additional freeze-thaw cycle. Please note the logarithmic scaling

We investigated the inter-assay variability of Aβ-coated SiNaPs, IQC samples, and 13 fecal samples in two different assays (Fig. 4). All three targets showed high comparability, indicated by Spearman’s coefficient of correlations (ρ: 1.0 for Aβ-coated SiNaPs and IQC samples and ρ: 0.929 (*p*-value: 8.63 × 10^− 4^) for fecal samples).

### Aβ aggregates are present in fecal samples and are elevated in AD patients

After completion of preanalytical and selectivity studies, a proof-of-concept study was performed using dilution series of Aβ-coated SiNaPs for calibration and LoD calculation, a dilution series of IQC samples and 57 fecal samples comprising two diagnostic groups (Table 1). In detail, we quantified fecal Aβ aggregate concentrations of HC subjects having no subjective cognitive decline (*n* = 31) and patients diagnosed with clinical AD (*n* = 26). Because the data mainly did not show normal distribution (Table S2), we performed statistical analysis using non-parametric tests like the Mann − Whitney U test or Spearman correlation.

**Table 1:** Demographic and clinical information on AD patients and HC that donated fecal samples

Characteristics	AD	HC
Number	26	31
Female	42.3%	64.5%
Age [years ± SD]	71.1 ± 8.6	49.2 ± 16.4
Bristol scale [score ± SD]	4.4 ± 0.9	5.1 ± 0.8

^a^Abbreviations: AD, Alzheimer’s disease; HC, healthy controls; SD, standard deviation

**Fig. 5:**
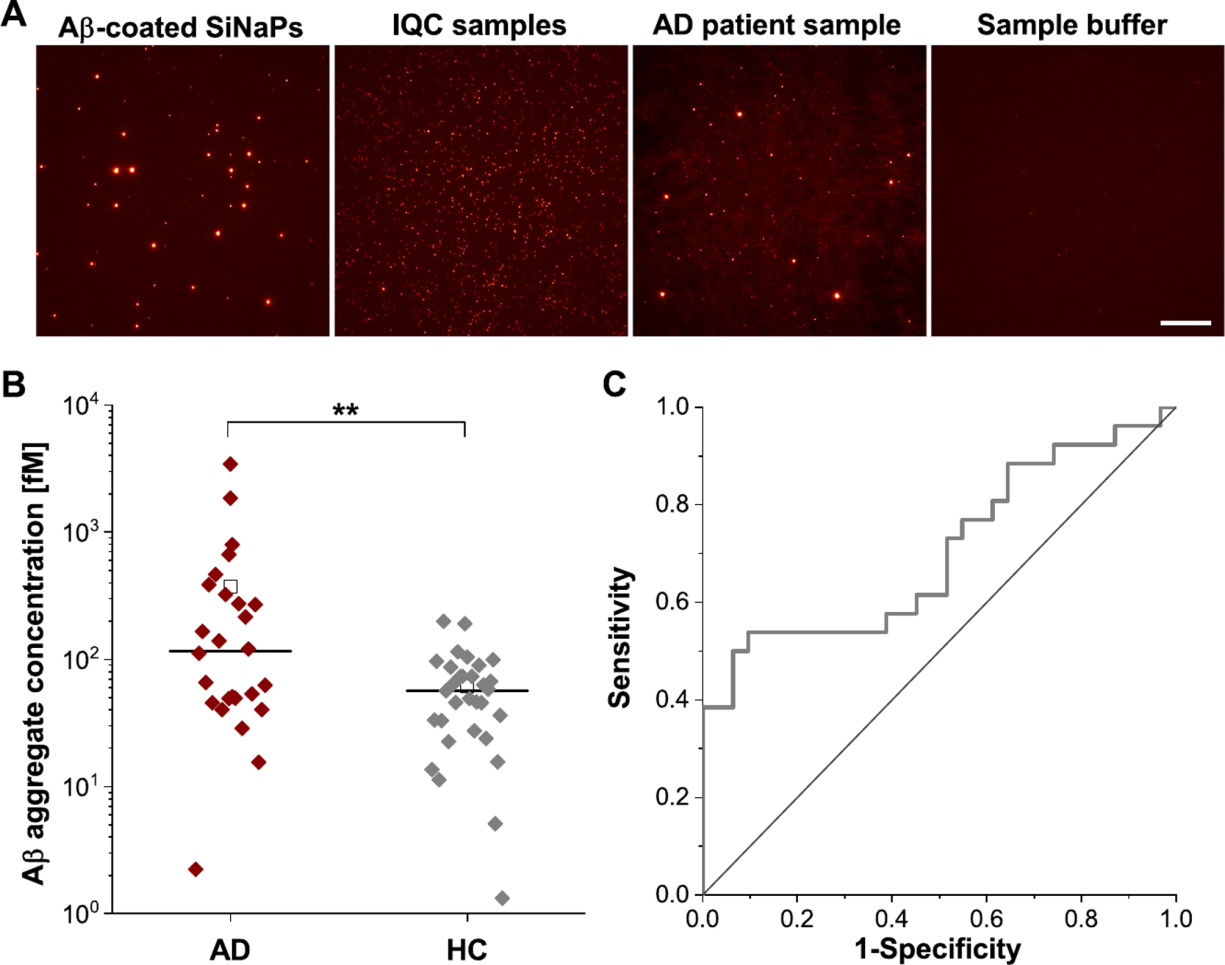
Representative TIRFM images, molar particle concentration of fecal Aβ aggregates and receiver operating characteristic. (**A**) Representative TIRFM images for the red channel (IC16-CF633, excitation 635 nm, emission 705 nm, exposure time 1000 ms, gain 1000) of 1 pM Aβ-coated SiNaPs, 100 pM synthetic Aβ1 − 42 oligomers (based on total Aβ concentration), fecal sample (AD patient) and sample buffer control. For better illustration of 14-bit images, color and contrast were adjusted using ImageJ software (colormap: red hot, contrast: maximum grayscale value 8000). Scale bar: 20 μm. (**B**) Concentrations of fecal Aβ aggregates of AD patients were significantly elevated with a *p*-value of 0.009 compared to HC. Significant differences between both cohorts were calculated with a Mann − Whitney U test (**p: ≤ 0.01). Please note the logarithmic scaling (line = median, square = mean). (**C**) In receiver operating characteristic (ROC) analysis, discrimination of AD patients versus HC showed a specificity of 90.3% and a sensitivity of 53.8% with an AUC of 0.703

Due to the high number of assay points, the measurements were performed on two 384-well microtiter plates (experiment 1: 48 samples, experiment 2: 9 samples), each carrying both a dilution series of Aβ-coated SiNaPs for calibration and LoD calculation and a dilution series of IQC samples, respectively. We determined a femtomolar mean LoD of 1.68 fM for Aβ-coated SiNaPs, indicating high analytical sensitivity. The mean CV% for Aβ-coated SiNaPs was 13.4% and 11.1% for IQC samples. A mean intra-assay variability of 18.7% was observed for fecal samples (for individual results of each experiment see Table S3). Representative TIRFM images of Aβ-coated SiNaPs, IQC sample, patient sample and the sample buffer control are shown in Fig. 5A.

Using Aβ-coated SiNaPs standards, pixel counts of fecal samples were calibrated into molar particle concentrations (Table S4). We determined Aβ aggregate concentrations ranging from 1.3 fM − 3.4 pM (Fig. 5B). Despite a substantial overlap between both groups, Aβ aggregate levels of AD patients were significantly elevated (*p*-value: 0.009). ROC curve were determined to evaluate the use of fecal Aβ aggregates as a diagnostic biomarker (Fig. 5C). Discrimination of AD patients versus HC showed a specificity of 90.3% and a sensitivity of 53.8% with an AUC of 0.703.

Since HC samples were collected anonymously, we could not match the sample with the demographic information of the respective donor. Thus, only the mean age and proportion of female donors was calculated. Therefore, a direct correlation to the determined fecal Aβ aggregate concentration could only be established for the AD cohort. Since we are aware that the samples used from HC do not match with samples of AD patients (Table 1), we investigated for the AD cohort whether fecal Aβ aggregate concentrations might be influenced by age or sex. However, no correlation between AD patients’ age and Aβ aggregate concentration was found using Spearman correlation (ρ: 0.163, *p*-value: 0.426). In addition, no difference was found in Aβ aggregate concentrations in fecal samples from male and female AD patients using a two-sided Mann − Whitney U test with a confidence interval of 5% (*p*-value: 0.959).

### Aβ aggregate quantification is not affected by sample consistency or endogenous substances

To investigate whether the feces consistency, indicated by Bristol scale, affects the measured fecal Aβ aggregate concentrations, we performed a Spearman correlation for all 57 samples. Here, we did not find a significant correlation, indicating that the quantification was not affected by feces consistency (ρ: −0.176, *p*-value: 0.191). Since we found significant differences in feces consistency between AD patients and HC (*p*-value: 0.003, Table 1), we performed additional Spearman correlation for HC and AD patient groups separately. Here, we also did not find any correlation between the Bristol scale and the signals of fecal Aβ-aggregate (AD patients: ρ: −0.087, *p*-value: 0.672; HC: ρ: 0.073, *p*-value: 0.696). Spearman correlation between fecal albumin, hemoglobin, calprotectin, IgA, bile acid, α-1-antitrypsin, lipids, and fecal Aβ aggregate concentrations were investigated to assess interfering effects. As shown in Table S5, Spearman correlation coefficients between − 0.38 and + 0.36 were observed. However, since they were not significant, only minute interfering effects of endogenous substances on quantification can be assumed. In addition, we investigated whether the levels of all seven biomarkers were different between AD patients and HC. As shown in Table S6, no significant differences between both cohorts were observed.

## Discussion

AD is the most common age-related cause of dementia and among the most critical public health problems in industrialized countries due to increasing life expectancy [47]. In AD pathology, small soluble Aβ oligomers are the most toxic Aβ species that damage neurons and compromise synaptic function. Aβ oligomers form probably decades before clinical symptoms of AD manifest in humans. We hypothesized that clearance mechanisms are likely to exist to protect the brain from toxic effects. The deposition of Aβ oligomers into amyloid plaques is likely just one clearance mechanism, while the sequestration of Aβ oligomers out of the brain could be another mechanism [12, 13, 16]. Given that the liver plays a crucial role in detoxifying the blood, it is reasonable to assume that Aβ oligomers are sequestered from the bloodstream by the liver and then transported to the gut via bile. We and others have provided compelling evidence indicating that disturbances along the brain-gut-microbiota axis may substantially contribute to the pathogenesis of neurodegenerative diseases such as AD because gastrointestinal metabolic, endocrine, neuronal, and immunological pathways are critical for the maintenance of brain homeostasis [5, 6, 9–11]. Although the bidirectional communication between the brain and gut and its microbiome is not yet fully understood, it is clear that changes in the gut microbiome can induce an immune activation resulting in a systematic inflammation, which in turn may compromise the intestinal barrier (leaky gut) and the blood-brain barrier [5, 6, 10, 11, 15]. Combined with a dysregulated Aβ homeostasis, brain-derived Aβ aggregates could thus directly enter the enteric nervous system by crossing the blood-brain barrier or by neuron-to-neuron, distal neuron spreading, or other cells like astrocytes, fibroblasts, microglia and immune system cells [5]. Conversely, Aβ species produced by enteric neurons [18] may also enter the brain. Moreover, due to the permeability of the intestinal barrier caused by systematic inflammation, it can be assumed that Aβ aggregates in blood or originating in enteric neurons enter the intestinal lumen and are excreted with feces, as we have observed for α-synuclein aggregates in patients with isolated rapid eye movement sleep behavior disorder, a prodromal form of parkinsonism [28]. Besides, circulating Aβ is predominantly cleared by degradation in hepatocytes and secreted into the gut in bile [12–14, 16], which in turn could increase intestinal and fecal Aβ concentrations. Initial studies have confirmed this association between AD and increased intestinal or fecal Aβ concentrations, irrespective of Aβ conformation [17–20]. One may assume, however, that only Aβ species that are resistant to proteases in the gut may become observable in feces.

The clinical assessment of AD is supported by neurological evaluation, imaging, and biomarkers in patients’ CSF [1, 4]. Due to the invasiveness and burden of a lumbar puncture on patients, CSF is not routinely collected [48, 49]. Identifying non-invasive biomarkers that can be used for sensitive detection of AD years or even decades before clinical onset is of utmost importance [22, 50]. Therefore, we used sFIDA technology to verify whether Aβ aggregates exist in feces and, more importantly, whether Aβ aggregate concentrations in fecal samples of HC and AD differ.

As this was a pilot project to use sFIDA for the quantification of fecal Aβ aggregates, we first had to overcome several preanalytical hurdles due to a complex sample matrix in addition to already existing challenges of oligomer-based diagnostics. Because the fecal samples must be processed before they are subjected to sFIDA, we first established a reproducible homogenization procedure. Various homogenization methods for fecal samples are described in the literature, with the required sample quantity being determined either by weighing or using tubes with an integral dosing system [51–55]. For our study, we opted to use Simplix tubes, which facilitated simple, clean, and efficient sample handling, and accurate dosing, as confirmed by our results.

The easiest way to overcome matrix effects in immunoassays is to dilute samples in a dilution buffer that ensures high discrimination between negative and positive samples [56]. In this study, a 1:5 dilution of the fecal homogenates was found optimal (data not shown) and reproducible in sFIDA assay development. Furthermore, as dilution linearity or parallelism, respectively, were observed for Aβ-coated SiNaPs, IQC sample, and fecal samples, we can exclude strong interferences due to, e.g., heterophilic antibodies as these are typically reflected in insufficient dilution linearity [42]. Therefore, samples containing high levels of endogenous fecal Aβ can be diluted within a linear range, and still yield reliable outcomes.

Chemical, microbial, and physical factors influence an analyte’s stability and measurable concentration in a complex sample and can significantly falsify a measurement [43, 45]. Because our thermostability study showed that incubation at temperatures above 4 °C did affect sample stability, we adjusted the sFIDA procedure for fecal analysis accordingly. In addition, we have developed a homogenization buffer, which combined with the homogenization process, leads to stable Aβ aggregates in feces, even when they are exposed to several freeze-thaw cycles. In contrast, crude samples reacted to freeze-thaw cycles with a signal reduction. These results are similar to those reported for CSF, where the stability of Aβ42 was analyzed, and a signal loss of 20% was observed after three freeze-thaw cycles [46, 57]. Especially, the signal of Aβ aggregates was further reduced with increasing cycle numbers, which is in accordance with our study in the case of crude fecal samples.

Despite complex matrix, using a suitable homogenization and sample dilution buffer ensured a high consistency within the sample replicates implicated by low intra-assay variability. Furthermore, Aβ-coated SiNaPs, IQC sample and fecal samples demonstrated low inter-assay variability, indicating a highly precise assay. In particular, the comparability of both sample measurements of the inter-assay study should be emphasized since the homogenates used for the second measurement were stored at − 80 °C for nine months. Thus, besides sufficient stability against freeze-thaw cycles, the homogenization buffer also allows aggregate stability over an extended period. Additionally, the sFIDA assay displayed a high level of selectivity for fecal Aβ aggregates.

After preanalytical and analytical validation, we determined the Aβ aggregate concentrations in the feces of AD patients and HC. All feces contained Aβ aggregates in the femtomolar range, a few samples even above. Although it is not clear whether the Aβ aggregates found in feces have been secreted by the liver/bile system or have been directly secreted into the gut, e.g., from neurons of the enteric system, this study supports the presence of clearance mechanisms that reduce Aβ oligomer concentration in the body. Here, we demonstrated that sFIDA is suitable to measure low femtomolar (1.3 fM) to low picomolar (3.4 pM) concentrations of Aβ aggregates in fecal samples, which is slightly higher than the previously reported concentration range of Aβ aggregates in CSF (aM-fM) [58, 59]. We also demonstrated that fecal samples of AD patients with proven amyloid-positivity in the CSF or brain showed significantly elevated levels of Aβ aggregates compared to HC, which has been previously shown for Aβ aggregate concentrations in CSF [27, 58–62]. Due to a high specificity for aggregated Aβ species, the sFIDA assay developed in the present study could discriminate between fecal samples from HC and AD patients with a specificity of 90.3%. Because potential clearance mechanisms can be expected to yield increased Aβ aggregate concentrations also in (still) HC, not surprisingly, we observed an overlap between both cohorts, resulting in an assay sensitivity of only 54%, which limits the clinical use at the current stage of development. In this context, longitudinal samples may help to determine possible changes in the fecal Aβ aggregate concentration before and during disease. The correlation between fecal Aβ aggregate concentration and clinical symptoms within the AD cohort could not be thoroughly evaluated in this study, as it only involved four patients with dementia and 22 with MCI. However, if future studies indeed confirm a correlation between fecal Aβ aggregate concentrations and cognitive abilities, it will represent a significant advancement toward non-invasive early detection of AD.

**Fig. 6:**
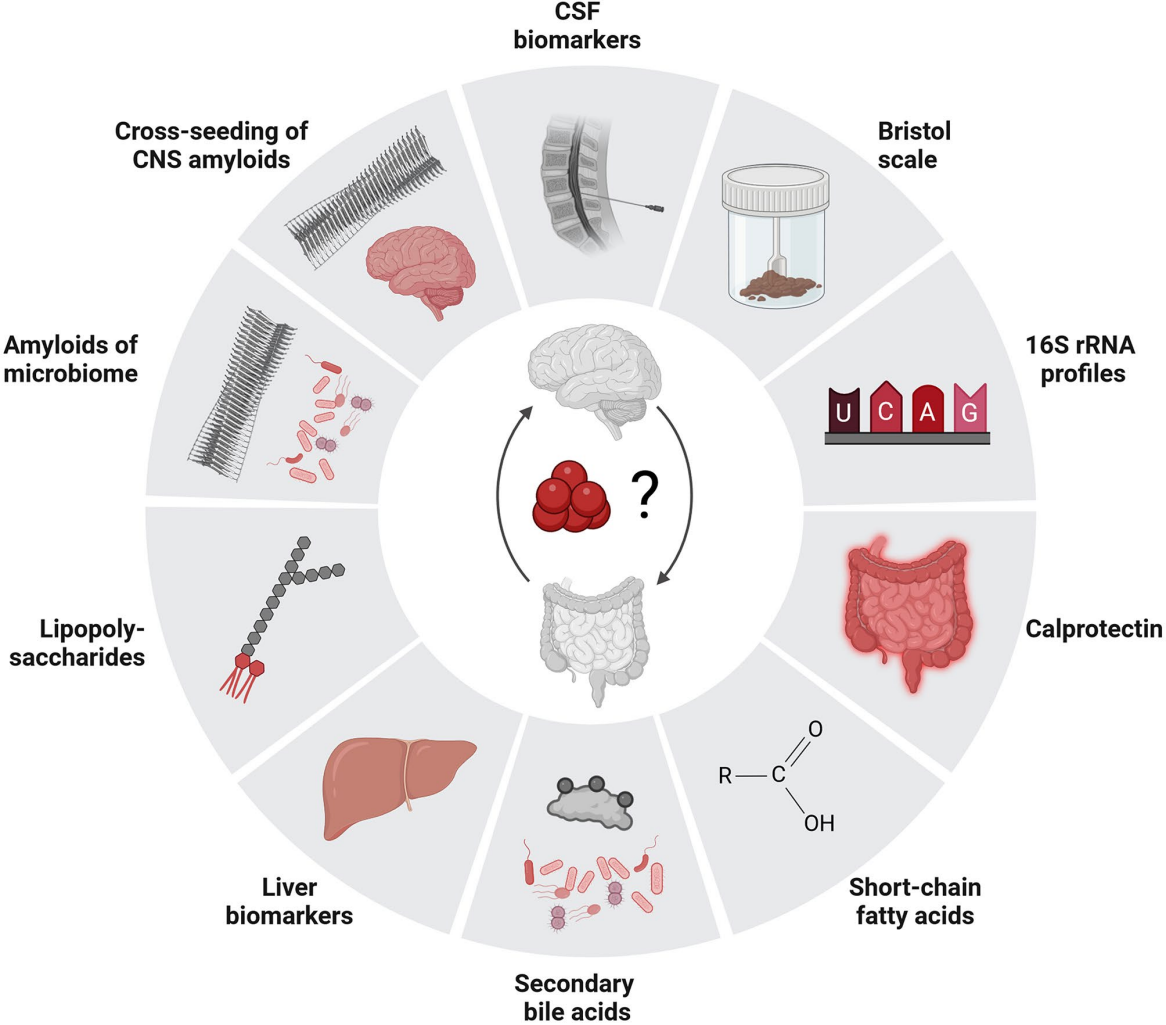
Association of fecal Aβ aggregates with additional biomarkers affecting AD pathology. The combination of fecal Aβ aggregates with further biomarkers may provide new insights into mechanism of brain-gut-microbiota axis and AD pathogenesis. Therefore, in addition to CSF biomarkers (Aβ40, Aβ42, phosphorylated and total Tau) and Bristol scale, 16 S rRNA profiles, fecal calprotectin, short-chain fatty acids, secondary bile acids, liver biomarkers, and lipopolysaccharides should be determined in the future. Because amyloids produced by gut microbiome share similarities in tertiary structure with CNS amyloids, they may act in a prion-like manner and induce misfolding, aggregation, and deposition of Aβ and may cross-seed with neuronal amyloids once they have entered the brain due to increased permeability of the blood-brain barrier. Created with BioRender.com

Since this is a proof-of-concept study, there are certain limitations to our findings, primarily due to the restricted availability of samples, resulting in small sample sizes. While we could demonstrate statistically significant (*p* < 0.01) elevation of fecal Aβ aggregates in AD vs. HC, it is crucial to replicate this result in larger validation studies employing independent cohorts. While in this study we did not observe any correlation between the age and Aβ aggregate concentration, we acknowledge that the samples obtained from HC were not matched in age with those from AD patients, which should be addressed in future work. Additionally, it would be intriguing to explore the link of fecal Aβ aggregates with additional biomarkers related to AD pathology, gut microbiota, and liver function (Fig. 6). Additional data on these biomarkers in combination with a larger cohort of patients across the AD continuum have the potential to enhance our comprehension of the fundamental disease pathology and enable early diagnosis at a stage when clearance mechanisms may be starting to malfunction.

## Conclusion

In conclusion, we developed a sFIDA assay for the quantitation of fecal Aβ aggregates, showing high reproducibility of preanalytical procedures, high analytical sensitivity, and specificity. In this work we delivered a proof-of-concept study, that Aβ aggregate are present in human feces and that AD patients exhibited elevated levels of fecal Aβ aggregates compared to HC. Future studies will need to confirm our results with more extensive cohort of participants, encompassing various AD stages, along with longitudinal samples and more comprehensive biomarker analysis. Finally, this work underscores the promising potential of fecal Aβ aggregates as a non-invasive biomarker for AD from which clinical routine and the development of therapeutic interventions might benefit in the future.

## Electronic supplementary material

Below is the link to the electronic supplementary material.

Supplementary Material 1

## Data Availability

All data generated or analysed during this study are included in this published article and its Supplementary Information. For image data analysis, we used the sFIDAta software application, which can be made available upon request.

## Abbreviations

Aβ: Amyloid β
AD: Alzheimer’s disease
ADL: Abilities of daily living
AF: Autofluorescence control
APTES: 3aminopropyl(triethoxysilane)
AUC: Area under the curve
BC: Blank control
BSA: Bovine serum albumin
CC: Capture control
CSF: Cerebrospinal fluid
CV%: Coefficient of variation
DMSO: Dimethyl sulfoxide
EDC: 1-ethyl-3-(3-dimethylaminopropyl)carbodiimide
HC: healthy controls
HFIP: 1,1,1,3,3,3-hexafluoro-2-propanol
IQC: Internal quality control
IVDs: In-vitro diagnostics
LoD: Limit of detection
MCI: Mild cognitive impairment
MES: 2-(N-morpholino)ethanesulfonic acid
MIHA: Maleimido hexanoic acid
NHS: N-hydroxysuccinimid
OD: Optical density
PBS: 10× phosphate-buffered saline
ROC: Receiver operating characteristic
SD: Standard deviation
sFIDA: Surface-based fluorescence intensity distribution analysis
SiNaP: Silica nanoparticle
TBS: 1× Tris-buffered saline
TBST: 1× Tris-buffered saline containing 0.05% Tween20
TIRFM: Total internal reflection fluorescence microscopy

## References

[1] Jack CR Jr, Bennett DA, Blennow K, Carrillo MC, Dunn B, Haeberlein SB, et al. NIA-AA research framework: toward a biological definition of Alzheimer’s disease. Alzheimers Dement. 2018;14(4):535–62. 10.1016/j.jalz.2018.02.018 PMC5958625 29653606

[2] Brown MR, Radford SE, Hewitt EW. Modulation of β-Amyloid fibril formation in Alzheimer’s disease by Microglia and infection. Front Mol Neurosci. 2020;13:609073. 33324164 10.3389/fnmol.2020.609073 PMC7725705

[3] Hampel H, Hardy J, Blennow K, Chen C, Perry G, Kim SH, et al. The amyloid-β pathway in Alzheimer’s disease. Mol Psychiatry. 2021;26(10):5481–503. 10.1038/s41380-021-01249-0 PMC8758495 34456336

[4] Kulenkampff K, Wolf Perez AM, Sormanni P, Habchi J, Vendruscolo M. Quantifying misfolded protein oligomers as drug targets and biomarkers in Alzheimer and Parkinson diseases. Nat Reviews Chem. 2021;5(4):277–94. 10.1038/s41570-021-00254-9 37117282

[5] Kowalski K, Mulak A. Brain-gut-microbiota Axis in Alzheimer’s Disease. J Neurogastroenterol Motil. 2019;25(1):48–60. 30646475 10.5056/jnm18087 PMC6326209

[6] Ferreiro AL, et al. Gut microbiome composition may be an indicator of preclinical Alzheimer’s disease. Sci Transl Med. 2023;15(700):eabo2984. 37315112 10.1126/scitranslmed.abo2984 PMC10680783

[7] Varesi A et al. The Potential Role of Gut Microbiota in Alzheimer’s Disease: From Diagnosis to Treatment. Nutrients, 2022. 14(3). 10.3390/nu14030668 PMC8840394 35277027

[8] Laske C, et al. Signature of Alzheimer’s Disease in Intestinal Microbiome: results from the AlzBiom Study. Front Neurosci. 2022;16:792996. 35516807 10.3389/fnins.2022.792996 PMC9063165

[9] Sun M, Ma K, Wen J, Wang G, Zhang C, Li Q, et al. A review of the brain-gut-microbiome axis and the potential role of microbiota in Alzheimer’s disease. J Alzheimers Dis. 2020;73(3):849–65. 10.3233/JAD-190872 31884474

[10] Leblhuber F, et al. Elevated fecal calprotectin in patients with Alzheimer’s dementia indicates leaky gut. J Neural Transm. 2015;122(9):1319–22. 25680441 10.1007/s00702-015-1381-9

[11] Fink A, Doblhammer G, Tamgüney G. Recurring gastrointestinal infections increase the risk of Dementia. J Alzheimers Dis. 2021;84:797–806. 34602468 10.3233/JAD-210316 PMC8673498

[12] Cheng Y, Tian DY, Wang YJ. Peripheral clearance of brain-derived Aβ in Alzheimer’s disease: pathophysiology and therapeutic perspectives. Transl Neurodegener. 2020;9(1):16. 32381118 10.1186/s40035-020-00195-1 PMC7204069

[13] Wang J, Gu BJ, Masters CL, Wang YJ. A systemic view of Alzheimer disease - insights from amyloid-β metabolism beyond the brain. Nat Rev Neurol. 2017;13(10):612–23. 10.1038/nrneurol.2017.111 28960209

[14] Ullah R, et al. Abnormal amyloid beta metabolism in systemic abnormalities and Alzheimer’s pathology: insights and therapeutic approaches from periphery. Ageing Res Rev. 2021;71:101451. 34450351 10.1016/j.arr.2021.101451

[15] Jiang C, et al. The gut microbiota and Alzheimer’s Disease. J Alzheimers Dis. 2017;58(1):1–15. 28372330 10.3233/JAD-161141

[16] Wu S, Hu L, Lin J, Li K, Ye S, Zhu S, et al. Excretion of amyloid-β in the gastrointestinal tract and regulation by the gut microbiota. J Alzheimers Dis. 2022;90:1153–62. 10.3233/JAD-220705 36214002

[17] Pellegrini C et al. Prodromal Intestinal Events in Alzheimer’s Disease (AD): Colonic Dysmotility and Inflammation Are Associated with Enteric AD-Related Protein Deposition. Int J Mol Sci, 2020. 21(10). 10.3390/ijms21103523 PMC7278916 32429301

[18] Manocha GD, Floden AM, Miller NM, Smith AJ, Nagamoto-Combs K, Saito T, et al. Temporal progression of Alzheimer’s disease in brains and intestines of transgenic mice. Neurobiol Aging. 2019;81:166–76. 10.1016/j.neurobiolaging.2019.05.025 PMC6732235 31284126

[19] Puig KL, Manocha GD, Combs CK. Amyloid precursor protein mediated changes in intestinal epithelial phenotype in vitro. PLoS ONE. 2015;10(3):e0119534. 25742317 10.1371/journal.pone.0119534 PMC4351204

[20] Joachim CL, Mori H, Selkoe DJ. Amyloid beta-protein deposition in tissues other than brain in Alzheimer’s disease. Nature. 1989;341(6239):226–30. 2528696 10.1038/341226a0

[21] Willbold D, Strodel B, Schröder GF, Hoyer W, Heise H. Amyloid-type protein aggregation and prion-like properties of amyloids. Chem Rev. 2021;121(13):8285–307. 10.1021/acs.chemrev.1c00196 34137605

[22] Kulawik A, Heise H, Zafiu C, Willbold D, Bannach O. Advancements of the sFIDA method for oligomer-based diagnostics of neurodegenerative diseases. FEBS Lett. 2018;592(4):516–34. 10.1002/1873-3468.12983 29360176

[23] Herrmann Y, Kulawik A, Kühbach K, Hülsemann M, Peters L, Bujnicki T, et al. sFIDA automation yields sub-femtomolar limit of detection for Aβ aggregates in body fluids. Clin Biochem. 2017;50(4–5):244–7. 10.1016/j.clinbiochem.2016.11.001 27823959

[24] Hülsemann M, et al. Biofunctionalized silica nanoparticles: standards in Amyloid-β oligomer-based diagnosis of Alzheimer’s Disease. J Alzheimers Dis. 2016;54(1):79–88. 27472876 10.3233/JAD-160253

[25] Pils M, Dybala A, Rehn F, Blömeke L, Bujnicki T, Kraemer-Schulien V, et al. Development and implementation of an internal quality control sample to standardize oligomer-based diagnostics of Alzheimer’s disease. Diagnostics. 2023;13(10):1702. 10.3390/diagnostics13101702 PMC10217173 37238187

[26] Blömeke L, et al. Quantitative detection of α-Synuclein and tau oligomers and other aggregates by digital single particle counting. NPJ Parkinsons Dis. 2022;8(1):68. 35655068 10.1038/s41531-022-00330-x PMC9163356

[27] Wang-Dietrich L, et al. The amyloid-β oligomer count in cerebrospinal fluid is a biomarker for Alzheimer’s disease. J Alzheimers Dis. 2013;34(4):985–94. 23313925 10.3233/JAD-122047

[28] Schaffrath A, et al. Patients with isolated REM-sleep behavior disorder have elevated levels of alpha-synuclein aggregates in stool. NPJ Parkinsons Dis. 2023;9(1):14. 36732520 10.1038/s41531-023-00458-4 PMC9895074

[29] Kass B, et al. Aβ oligomer concentration in mouse and human brain and its drug-induced reduction ex vivo. Cell Rep Med. 2022;3(5):100630. 35584626 10.1016/j.xcrm.2022.100630 PMC9133466

[30] Kalbe E, et al. DemTect: a new, sensitive cognitive screening test to support the diagnosis of mild cognitive impairment and early dementia. Int J Geriatr Psychiatry. 2004;19(2):136–43. 14758579 10.1002/gps.1042

[31] Beck AT, et al. Comparison of Beck Depression inventories -IA and -II in psychiatric outpatients. J Pers Assess. 1996;67(3):588–97. 8991972 10.1207/s15327752jpa6703_13

[32] Crook TH 3rd, Feher EP, Larrabee GJ. Assessment of memory complaint in age-associated memory impairment: the MAC-Q. Int Psychogeriatr. 1992;4(2):165–76. 1477304 10.1017/s1041610292000991

[33] Pfeffer RI, et al. Measurement of functional activities in older adults in the community. J Gerontol. 1982;37(3):323–9. 7069156 10.1093/geronj/37.3.323

[34] Albert MS, et al. The diagnosis of mild cognitive impairment due to Alzheimer’s disease: recommendations from the National Institute on Aging-Alzheimer’s Association workgroups on diagnostic guidelines for Alzheimer’s disease. Alzheimers Dement. 2011;7(3):270–9. 21514249 10.1016/j.jalz.2011.03.008 PMC3312027

[35] Lewis SJ, Heaton KW. Stool form scale as a useful guide to intestinal transit time. Scand J Gastroenterol. 1997;32(9):920–4. 9299672 10.3109/00365529709011203

[36] Dornieden S, et al. Characterization of a single-chain variable fragment recognizing a linear epitope of aβ: a biotechnical tool for studies on Alzheimer’s disease? PLoS ONE. 2013;8(3):e59820. 23555792 10.1371/journal.pone.0059820 PMC3608532

[37] Qian XH, Xie RY, Liu XL, Tang HD. Mechanisms of short-chain fatty acids derived from Gut microbiota in Alzheimer’s disease. Aging Dis. 2022;13(4):1252–66. 10.14336/AD.2021.1215 PMC9286902 35855330

[38] Vogt NM, et al. Gut microbiome alterations in Alzheimer’s disease. Sci Rep. 2017;7(1):13537. 29051531 10.1038/s41598-017-13601-y PMC5648830

[39] Vandeputte D, et al. Stool consistency is strongly associated with gut microbiota richness and composition, enterotypes and bacterial growth rates. Gut. 2016;65(1):57–62. 26069274 10.1136/gutjnl-2015-309618 PMC4717365

[40] Vanderstichele H, et al. Potential sources of interference on Abeta immunoassays in biological samples. Alzheimers Res Ther. 2012;4(5):39. 23082750 10.1186/alzrt142 PMC3580396

[41] Dimeski G. Interference testing. Clin Biochem Rev. 2008;29(Suppl 1):S43–8. 18852856 PMC2556582

[42] Park JY, Kricka LJ. Chap. 5*.3 - interferences in Immunoassay*. The Immunoassay Handbook (Fourth Edition). Oxford: Elsevier; 2013. pp. 403–16. D. Wild, Editor.

[43] Andreasson U, et al. A practical guide to Immunoassay Method Validation. Front Neurol. 2015;6:179. 26347708 10.3389/fneur.2015.00179 PMC4541289

[44] Armbruster DA, Pry T. Limit of blank, limit of detection and limit of quantitation. Clin Biochem Rev. 2008;29(Suppl 1):S49–52. 18852857 PMC2556583

[45] van de Merbel N, et al. Stability: recommendation for best practices and harmonization from the Global Bioanalysis Consortium Harmonization Team. Aaps j. 2014;16(3):392–9. 24550081 10.1208/s12248-014-9573-z PMC4012051

[46] Vanderstichele HM, et al. Optimized standard operating procedures for the analysis of Cerebrospinal Fluid Aβ42 and the ratios of Aβ isoforms using low protein binding tubes. J Alzheimers Dis. 2016;53(3):1121–32. 27258423 10.3233/JAD-160286 PMC4981898

[47] Salvadores N, et al. Detection of misfolded Aβ oligomers for sensitive biochemical diagnosis of Alzheimer’s disease. Cell Rep. 2014;7(1):261–8. 24656814 10.1016/j.celrep.2014.02.031

[48] Teunissen CE, et al. Blood-based biomarkers for Alzheimer’s disease: towards clinical implementation. Lancet Neurol. 2022;21(1):66–77. 34838239 10.1016/S1474-4422(21)00361-6

[49] Paraskevaidi M et al. Diagnostic Biomarkers for Alzheimer’s Disease Using Non-Invasive Specimens. J Clin Med, 2020. 9(6). 10.3390/jcm9061673 PMC7356561 32492907

[50] Lewczuk P, Łukaszewicz-Zając M, Mroczko P, Kornhuber J. Clinical significance of fluid biomarkers in Alzheimer’s disease. Pharmacol Rep. 2020;72(3):528–42. 10.1007/s43440-020-00107-0 PMC7329803 32385624

[51] Phadia-AB. *Stool Extraction Kit plus / Stool Extraction Buffer plus, instruction for use: 200/250/2500/5000-6665-020 / UK, version 2021-09-14.*. 2021; Available on https://dfu.phadia.com/Data/Pdf/6130cb9570eec660e274806b.pdf [Accessed 23 January 2023].].

[52] ORGENTEC-Diagnostika-GmbH. Calprotectin, instruction for use: ORG 580_4, version ORG 580_IFU_EN_QM140866_2022-03-08_4. 2022; Available on https://products.orgentec.com/pdfs/ifu/ORG%20580_IFU_EN_QM140866_2022-03-08_4.pdf [Accessed 23 January 2023].].

[53] Immundiagnostik-AG. *IDK Hemoglobin ELISA: For the in vitro determination of hemoglobin in stool, instruction for use: K 7816D, K7816D.20, version 2022-02-22.* 2022; Available on https://www.immundiagnostik.com/media/pages/testkits/k-7816d/9a63870b70-1663639307/k7816d_2022-02-22_haemoglobin.pdf [Accessed 23 January 2023].].

[54] Demeditec-Diagnostics-GmbH. *Calprotectin ELISA, instruction for use: DE849 V211014/DLB, version 2021-11-25.*. 2021; Available on https://www.demeditec.de/de/produkte/calprotectin-elisa-de849/ifu-de849-calprotectin-elisa-211125-e.pdf [Accessed 23 January 2023].].

[55] Bühlmann-Laboratories. Bühlmann Smart-Prep - Faecal sample preparation kit, Instruction for use: B-CAL-RD, version 2010-11-10. 2010; Available on https://www.buhlmannlabs.ch/wp-content/uploads/2015/01/B-CAL-RD_101110.pdf [Accessed 23 January 2023].].

[56] Minic R, Zivkovic I. *Optimization, Validation and Standardization of ELISA*. 2020.

[57] Schoonenboom NS, et al. Effects of processing and storage conditions on amyloid beta (1–42) and tau concentrations in cerebrospinal fluid: implications for use in clinical practice. Clin Chem. 2005;51(1):189–95. 15539465 10.1373/clinchem.2004.039735

[58] Yang T, et al. A highly sensitive novel immunoassay specifically detects low levels of soluble Aβ oligomers in human cerebrospinal fluid. Alzheimers Res Ther. 2015;7(1):14. 25802556 10.1186/s13195-015-0100-y PMC4369838

[59] Savage MJ, et al. A sensitive aβ oligomer assay discriminates Alzheimer’s and aged control cerebrospinal fluid. J Neurosci. 2014;34(8):2884–97. 24553930 10.1523/JNEUROSCI.1675-13.2014 PMC6608513

[60] Herskovits AZ, et al. A Luminex assay detects amyloid β oligomers in Alzheimer’s disease cerebrospinal fluid. PLoS ONE. 2013;8(7):e67898. 23844122 10.1371/journal.pone.0067898 PMC3699502

[61] Fukumoto H, et al. High-molecular-weight beta-amyloid oligomers are elevated in cerebrospinal fluid of Alzheimer patients. Faseb j. 2010;24(8):2716–26. 20339023 10.1096/fj.09-150359

[62] Hölttä M, et al. Evaluating amyloid-β oligomers in cerebrospinal fluid as a biomarker for Alzheimer’s disease. PLoS ONE. 2013;8(6):e66381. 23799095 10.1371/journal.pone.0066381 PMC3682966

