# Supplementary material for: Elevated Aβ aggregates in feces from Alzheimer’s disease patients: a proof-of-concept study
Source: Alzheimers Res Ther. 2024 Oct 14;16:223. doi: 10.1186/s13195-024-01597-3 (PMC11472473; doi:10.1186/s13195-024-01597-3)
Supplement: Supplementary file 1 — Supplementary Material 1 [file 13195_2024_1597_MOESM1_ESM.docx]

**Elevated Aβ aggregates in feces from**

**Alzheimer’s disease patients: a proof-of-concept study**

**Supplementary Information**

Marlene Pils, Alexandra Dybala, Anja Schaffrath, Fabian Rehn, Janine Kutzsche, Lara Blömeke, Markus Tusche, Pelin Özdüzenciler, Tuyen Bujnicki, Victoria Kraemer-Schulien,
Hannes Gramespacher, Maximilian H.T. Schmieschek, Michael T. Barbe, Oezguer A. Onur, Gereon R. Fink, Gültekin Tamgüney, Oliver Bannach and Dieter Willbold

# Supplementary Methods

## Homogenization of fecal samples for sFIDA analysis

In this study, we used Tris buffer (20 mM Tris and 250 mM NaCl, both AppliChem, Darmstadt, Germany, pH 8.3), which was previously applied to quantify Aβ aggregates in homogenized brain tissue using sFIDA technology [1]. As during homogenization release of proteases and phosphatases cannot be excluded, we added cOmplete EDTA-free protease inhibitor and PhosStop phosphatase inhibitor (both Roche, Basel, Switzerland) as described in the study of Kass et al. [1]. To reduce non-specific binding of Aβ aggregates to the homogenization tubes, we added 1% bovine serum albumin (BSA, AppliChem). Furthermore, the properties of glycerol (Carl Roth, Karlsruhe, Germany) were used to further stabilize the analyte, especially during repeated freezing and thawing [2], using a concentration of 5%. Because about a quarter of the solid fecal components comprises bacteria and other microorganisms that may still be viable [3, 4], we added NaN_3_ (AppliChem) at a concentration of 0.05%.

Various feces homogenization methods are described in the literature, with the required sample quantity being determined either by weighing or using tubes with an integral dosing system [5-9]. We decided to use such a dosing system, i.e., Simplix tubes (polypropylene, Gaudlitz, Coburg, Germany), allowing for simple, clean, and fast sample handling and accurate dosing. The homogenization procedure used in this study (Supplementary Figure 1) was adapted by combining already established instructions of *in-vitro* diagnostics (IVDs) using feces as sample matrix for the quantification of other biomarkers, e.g., fecal IgA, hemoglobin, or calprotectin [5, 6, 8, 9].

## Preanalytics

### Reproducibility of preanalytical procedures

Due to the complex and individually varying composition of feces, we tested the reproducibility of homogenization and sample dilution using three fecal samples with an initial low, intermediate, and high readout. To this end, six homogenates of each of the three samples were prepared, whereby the weight of the sample was additionally calculated based on the previously recorded empty weight (homogenization tube with buffer). Based on these data and using the reference weight of 17 mg according to the manufacturer's specifications of the Simplix tubes, the sample dosage's precision and trueness were calculated. Each homogenate was subjected to sFIDA assay in quadruplicates. In addition, the reproducibility of sample dilution was investigated by preparing four dilutions from one homogenate of each sample and assaying them also in quadruplicate within sFIDA. Afterward, pixel counts of individual replicates of each sample were normalized based on Eq.1. Values within the tolerance range of ± 25% indicated sufficient reproducibility.

1. $Normalized pixel count \left[ \% \right]= \frac{{pixel count}_{replicate x}}{{pixel count}_{mean all replicates}}\times100 \%$

### Dilution linearity

In this study, the dilution linearity of Aβ-coated SiNaPs and IQC in dilution buffer was investigated. Before calculating the dilution linearity of both targets, background correction was performed by subtracting the BC value from each value. Subsequently, the percent dilution linearity of each calibration or IQC sample was calculated using Eq.2. Dilution linearity was accepted when the mean percent dilution linearity was between 80−120%.

1. $Dilution linearity or parallelism \left[ \% \right]= \frac{observed concentration}{(previous observed value/dilution factor)}\times100 \%$

For investigating parallelism, two fecal samples with high endogenous concentrations of Aβ aggregates were homogenized and diluted 1:5 with sample dilution buffer. Subsequently, these samples were serially diluted four times by a factor of two, and all prepared dilutions were applied on sFIDA surface. Because parallelism and dilution linearity are conceptually similar [10], the percent parallelism of each dilution step was also calculated using Eq.2. In the case of fecal samples, acceptance criteria for the mean dilution linearity were increased up to ± 25%.

### Sample stability

To assess thermostability, three crude fecal samples with low, intermediate, and high readouts were stored at −20 °C, 4 °C, or 20 °C for 3, 6, or 18 h to simulate sample transport and bench top conditions, respectively. To this end, nine Simplix tubes were prefilled with the respective fecal sample, and the individual homogenization tubes were stored according to the storage condition mentioned above. At the end of each storage period, the aliquots were frozen at −80 °C. As a reference, another Simplix tube was prefilled with the corresponding sample and stored directly at −80 °C. On the day of the sFIDA experiment, all Simplix tubes were thawed simultaneously, the homogenization tubes were filled with homogenization buffer, and the samples were homogenized and diluted 1:5 with sample dilution buffer. Finally, all samples were measured by sFIDA, and the normalized pixel counts were calculated (Eq.1), where values within a tolerance range of ± 25% were accepted. Values outside the tolerance range indicated that sample stability was compromised.

For CSF samples, it is known that repeated thawing and refreezing can drastically reduce the measurable concentration of Aβ. In order to test whether similar effects can be observed in crude and already homogenized fecal samples, the effect of multiple freeze-thaw cycles (none, one, two, three, five, or seven) on the stability of the three samples was investigated according to Andreasson et al. [10]. To this end, six Simplix tubes each were prefilled with the respective crude sample and then stored at −80 °C. In contrast, for the investigation of fecal homogenates, homogenates of three samples were prepared at the beginning of the stability study, whereby the supernatant obtained after centrifugation was divided into six aliquots of 100 µL each. For this purpose, 1.5 mL polypropylene low-binding Eppendorf reaction tubes were used as containers, and all aliquots were frozen at −80 °C. Subsequently, the individual Simplix tubes and the fecal homogenates were thawed successively, while the reference aliquots did not undergo a thaw-freezing process during the study. After an incubation time of 2 h at RT, samples were frozen again for at least 12 h at −80 °C. At the end of the study period, all aliquots were thawed simultaneously and diluted 1:5 with sample buffer, while the crude fecal samples were first homogenized. The data acquisition and analysis of the image data were carried out analogously to the thermostability study.

### Assay selectivity

#### Assay selectivity – investigation of assay controls

To evaluate the selectivity of the sFIDA assay in detecting Aβ-coated SiNaPs (molar particle concentration of 10.26 pM), IQC samples (100 nM, Aβ monomer subunit concentration), and three fecal samples with intermediate to high readouts, we measured the percent signal reduction of capture, autofluorescence, and cross-reactivity control, and compared it to a standard assay setup. To exclude unspecific binding of Aβ to the blocking agent, a capture control (CC) was performed in which no capture antibody was added to the surface. The assay was performed without adding any fluorescent probes to exclude autofluorescence (AF) events from assay components or samples. In addition, we also tested the cross-reactivity of anti-α-synuclein antibodies (detection antibody 211) against the immobilized Aβ species. For each assay control setup, the ratio of observed and expected (standard assay setup) values and the percentage amount of reduced signal were calculated according to Eq.3. Values between 80−120% were accepted for Aβ-coated SiNaPs and IQC sample. In comparison, the tolerance range for fecal samples was increased up to ± 25%.

1. $Signal reduction \left[ \% \right]=(1-\frac{observed readout assay control}{readout reference} \times100\%)$

#### Assay selectivity – immunodepletion

Immunodepletion was performed on three fecal samples with intermediate to high readouts, Aβ-coated SiNaPs, and IQC samples to determine if the observed pixel counts were specifically attributed to Aβ aggregates and not to interfering fecal matrix components. To this end, the Nab228 antibody was covalently coated on the surface of carboxylated magnetic dynabeads (Invitrogen, Waltham, USA), as previously described by Blömeke et al. [11]. Briefly, the dynabeads were washed twice with 2.5 mM 2-(N-morpholino)ethanesulfonic acid (MES, pH 5.0, Carl Roth, Karlsruhe, Germany) before being applied to a magnet to remove the supernatant. Afterward, carboxy groups of the dynabeads were activated for 30 min at RT using 50 μg/mL EDC and 50 μg/mL NHS in MES, and the dynabeads were rewashed with MES and coated with Nab228 antibody to a concentration of 20 μg/mL dynabeads. The control dynabeads were coated with an α-synuclein-specific antibody 211 to investigate Aβ specificity of the depletion protocol. After antibody incubation for 1 h at RT, coated dynabeads were rewashed, and still activated carboxy groups were quenched with 50 mM ethanolamine in MES for 1 h at RT, followed by a final washing step. We applied 0.5 mg of antibody-coated dynabeads to the magnet for immunodepletion and removed the supernatant. 100 μL sample (10.26 pM Aβ-coated SiNaPs, 100 nM synthetic Aβ1−42 oligomers, and three fecal samples, which were diluted 1:5 in sample buffer before depletion) were added and incubated for 1 h at RT while rotating. After incubation, dynabeads were applied to the magnet again and the supernatant was transferred into a fresh tube and stored at −80 °C until sFIDA analyses. To assess assay selectivity, the efficiency of the immunodepletion was calculated according to Eq.3. The acceptance criteria were the same as for the assay control setup described above.

#### Assay selectivity – detection of Aβ using ELISA

To confirm whether Aβ species are present in human fecal samples, we used a human amyloid β (1−42) ELISA Assay kit (IBL International, Hamburg, Germany), where total Aβ (monomers and aggregates) was analyzed. Since the ELISA only includes plasma, CSF or cell culture supernatants as sample matrix, the use of fecal samples is not intended. Consequently, it cannot be assumed that the calibration of the optical density (OD) values in molar concentrations is still valid for fecal samples. However, it should at least be applicable for the qualitative detection of Aβ in the feces by comparing the OD values. We investigated two fecal samples (HC and AD donor) which were as follows:

Homogenized: For sample homogenization, 15 mg of each sample were transferred into a low binding Eppendorf tube, which was prefilled with 20 µL H_2_O containing protease inhibitor. After homogenization using vortex mixer, homogenates were incubated for 10 min at 95 °C at 600 rpm in order to free as many epitopes as possible. To separate insoluble digestive residues, homogenates were centrifuged for 5 min at 3000× *g*, 4 °C, and supernatants were used for ELISA analysis.

Precipitated: In another preparation approach, we eliminated and enriched Aβ from the sample matrix using immunoprecipitation. To this end, 1.5 g feces per donor were required to obtain 100 µL supernatant. Protein-G magnetic dynabeads (Invitrogen, Waltham, USA) were functionalized with IC16 antibody and homogenized samples were precipitated according to manufactures protocol. Afterward, Aβ was eluted using 10 mM acetic acid, resuspended in H_2_O containing protease inhibitor and supernatants were used for ELISA analysis.

Spiked + precipitated: To be sure that Aβ1−42 in stool samples can be determined by the applied ELISA assay, we spiked 100 pg/mL monomeric Aβ1−42 (Bachem, Bubendorf, Switzerland) to the homogenates prior to immunoprecipitation in a third preparation approach. In addition, we conducted a spike and recovery experiment to assess matrix effects. To this end, 100 pg/mL Aβ1−42 were spiked into assay buffer and into homogenized human fecal samples. The recovery rate in percent was calculated for spiked test samples by comparison to the measured recovery of spiked assay buffer control (reference) (see Eq. 4).

1. $\text{Recovery rate }\left[ \text{\%} \right]\text{=}\frac{\text{assay readout}_{\text{spiked samples}}}{\text{assay readout}_{\text{Reference}}}\text{× 100\%}$

The ELISA was performed according to manufactures protocol and OD values of the fecal samples of HC and AD were compared.

# Supplementary Figures


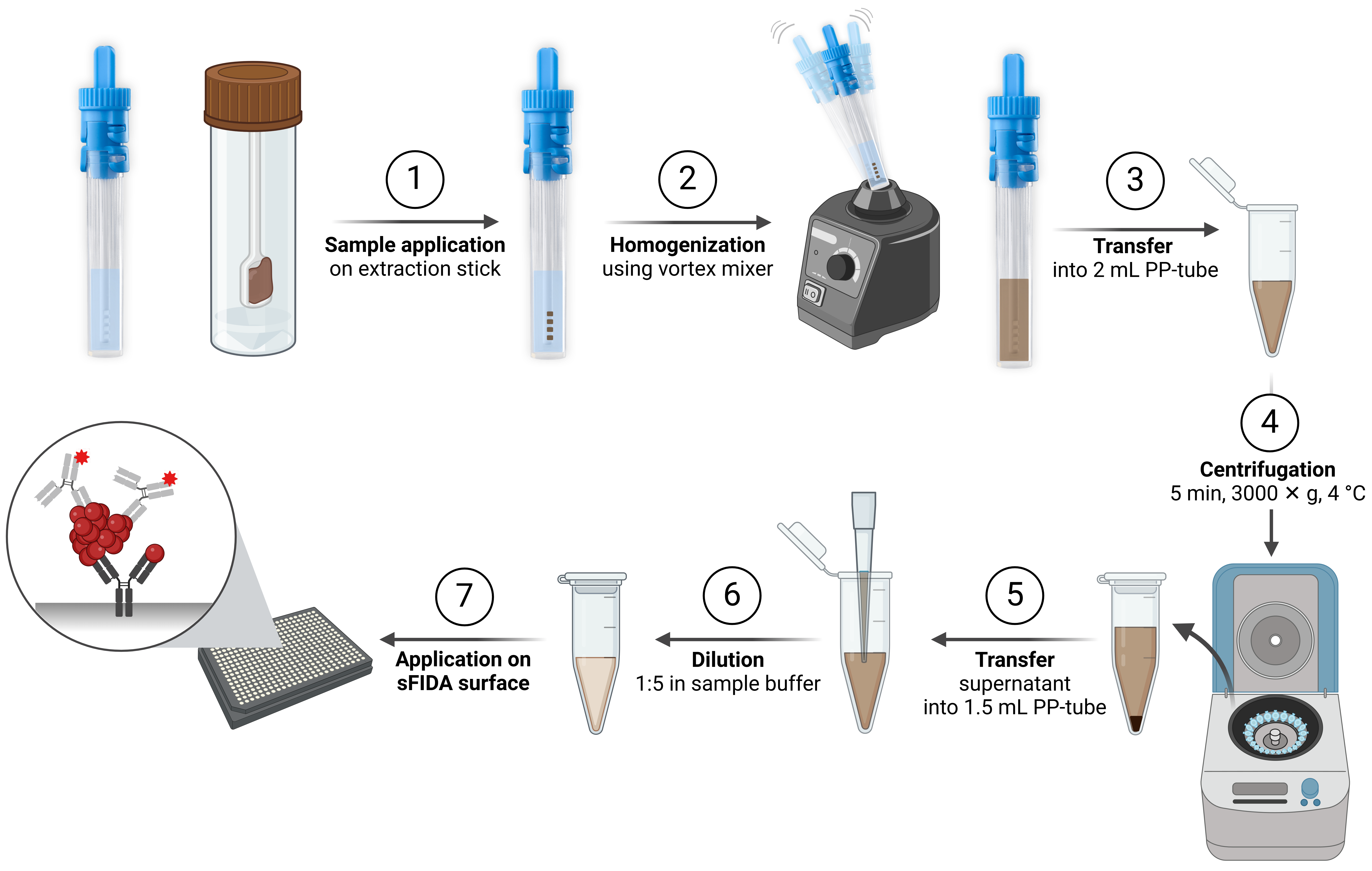


**Figure S1. Schematic illustration of the preanalytical processing of fecal samples prior sFIDA analysis, related to Methods.**

Step1: Simplix tubes were prefilled with cold 1300 µL homogenization buffer. Afterward, thawed fecal samples were applied on a sample stick, whereby the integrated dosing system ensured a sample weight of approximately 17 mg. Due to the liquid consistency, fecal samples with Bristol scale seven were weighted into Simplix tubes. Step 2: samples were homogenized completely using a vortex mixer and incubated for 10 min at RT. Step 3: homogenates were transferred into 2 mL polypropylene low-binding tubes (Eppendorf, Hamburg, Germany). Step 4: since dead bacteria can also show autofluorescence, these and solid components, e.g., undigested food residues, were mechanically separated from the liquid phase by centrifugation (5 min, 3000× g, 4 °C). Step 5: the supernatants were transferred into fresh 1.5 mL polypropylene low-binding tubes and stored at −80 °C before sFIDA analysis. Step 6: homogenates were diluted 1:5 in sample buffer (PBS containing 0.05% Tween, 0.095% NaN3 (AppliChem) and 0.5% BSA). Step 7: diluted homogenates were applied in fourfold determination on sFIDA surface where Aβ aggregates are immobilized by the capture antibody and labeled for microscopy using detection probes.


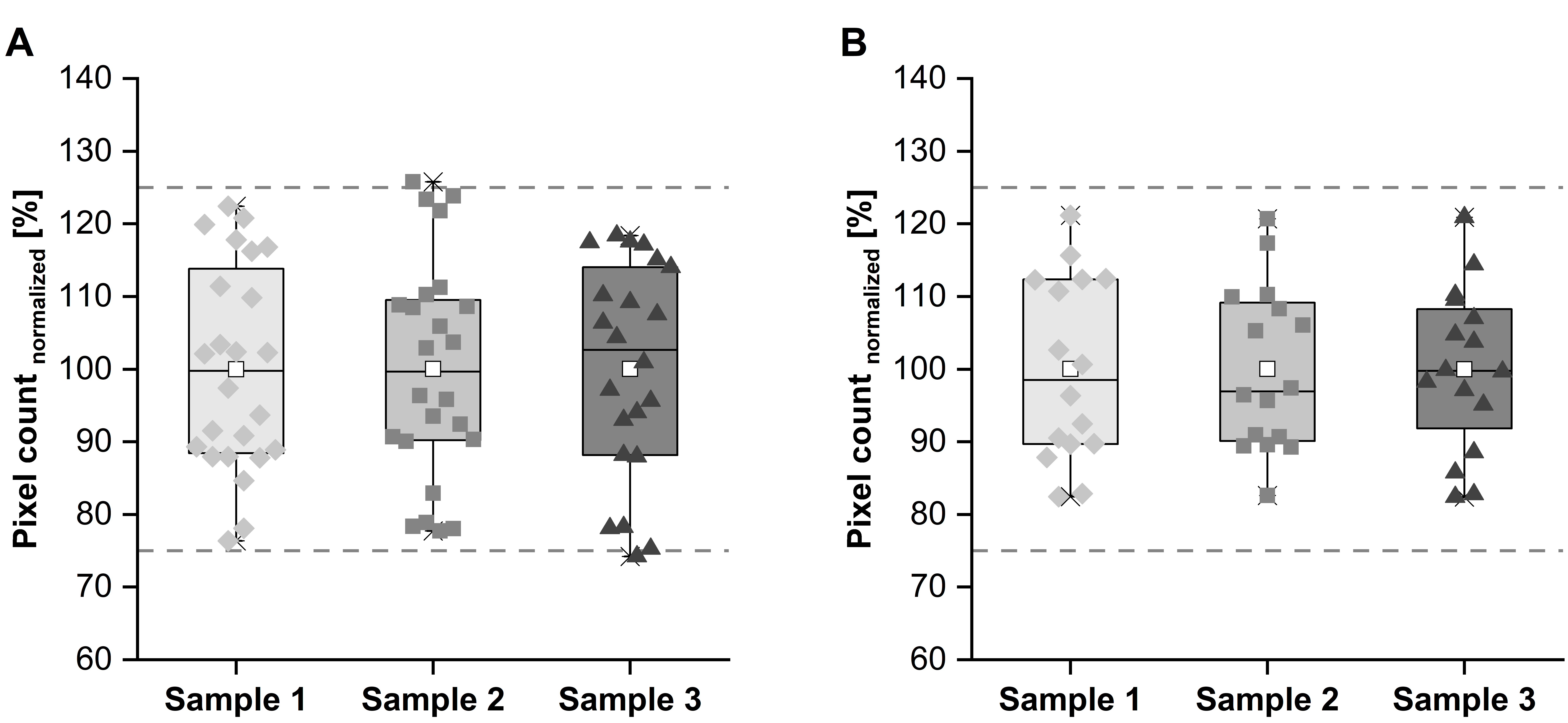


**Figure S2. Evaluation of the reproducibility of sample homogenization and dilution for three different fecal samples, related to Results.**

(**A**) To evaluate the reproducibility of sample homogenization, we prepared six homogenates from each of three different fecal samples. Each homogenate was diluted 1:5 and was measured by sFIDA in 4-fold replicates, and reproducibility was determined based on normalized pixel counts. In detail, the mean pixel count of the 24 data points was calculated. Afterward, the pixel count of each data point was divided by the mean value and multiplied by 100% according to Eq.1. The normalized pixel counts were illustrated as box plot. Consequently, there were 24 data points per box for each of the three samples (except for sample 3, where only 22 data points were available due to measurement artifacts in the affected wells). Intra-assay variances described by CV% of about 15% were recorded for all three samples confirming the reproducibility of the homogenization procedure. Based on documented sample weight, a precision of 98.6% and a trueness of 99.4% were calculated for the sample dosing using Simplix tubes. (**B**) We prepared a 1:5 dilution of each sample homogenate four times and subjected each dilution to sFIDA in quadruplicates. In general, we observed a high reproducibility with normalized pixel counts of the three fecal samples not exceeding the predefined tolerance range of ± 25% and showing low intra-assay variance (CV% of sample 1: 12.6%, samples 2 and 3: 11.3% each). For all replicates, a precision of 88.3% was determined, indicating a high reproducibility of the sample dilution (tolerance range 80−120%). Box plots include the median as a line and the mean of normalized pixel counts as a square. The tolerance range of ± 25% is depicted as dashed gray lines. sample 1 = diamonds, low readout; sample 2 = squares, intermediate readout, and sample 3 = triangles, high readout.


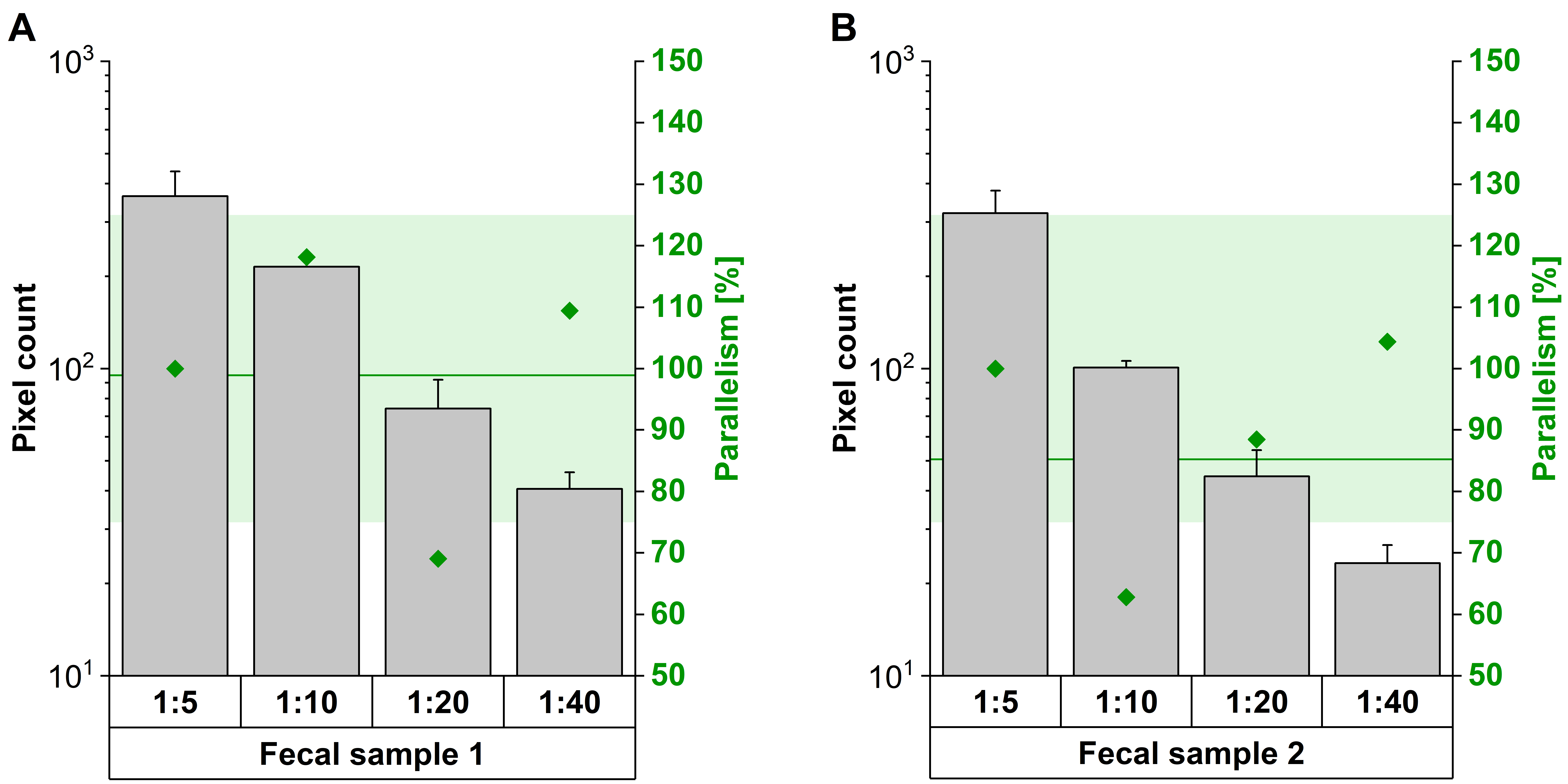


**Figure S3. Parallelism of endogenous Aβ aggregates in fecal sample 1 and sample 2, related to Results.**

We performed a parallelism study using two fecal samples (sample 1 (**A**), sample 2 (**B**) with high endogenous Aβ aggregate concentrations to test whether samples can be diluted linearly. The pixel counts (left y-axis, gray bars) and the calculated percent parallelism (dilution linearity, right y-axis, dark green diamonds) were plotted against the used sample dilution. The acceptance range for percent parallelism was 75−125%, indicated by light green background shading. Mean percent dilution linearities of 99.9%, for sample 1, and 85.2%, for sample 2, were observed, although two values exceeded the lower tolerance limit (1:20 dilution of sample 1, 1:10 dilution of sample 2). Thus, we can exclude strong interferences due to, e.g., heterophilic antibodies, which typically affect dilution linearity [12]. Consequently, samples with a high Aβ aggregate level can be diluted within a linear range and yield reliable results. Data are represented as mean with standard deviation.


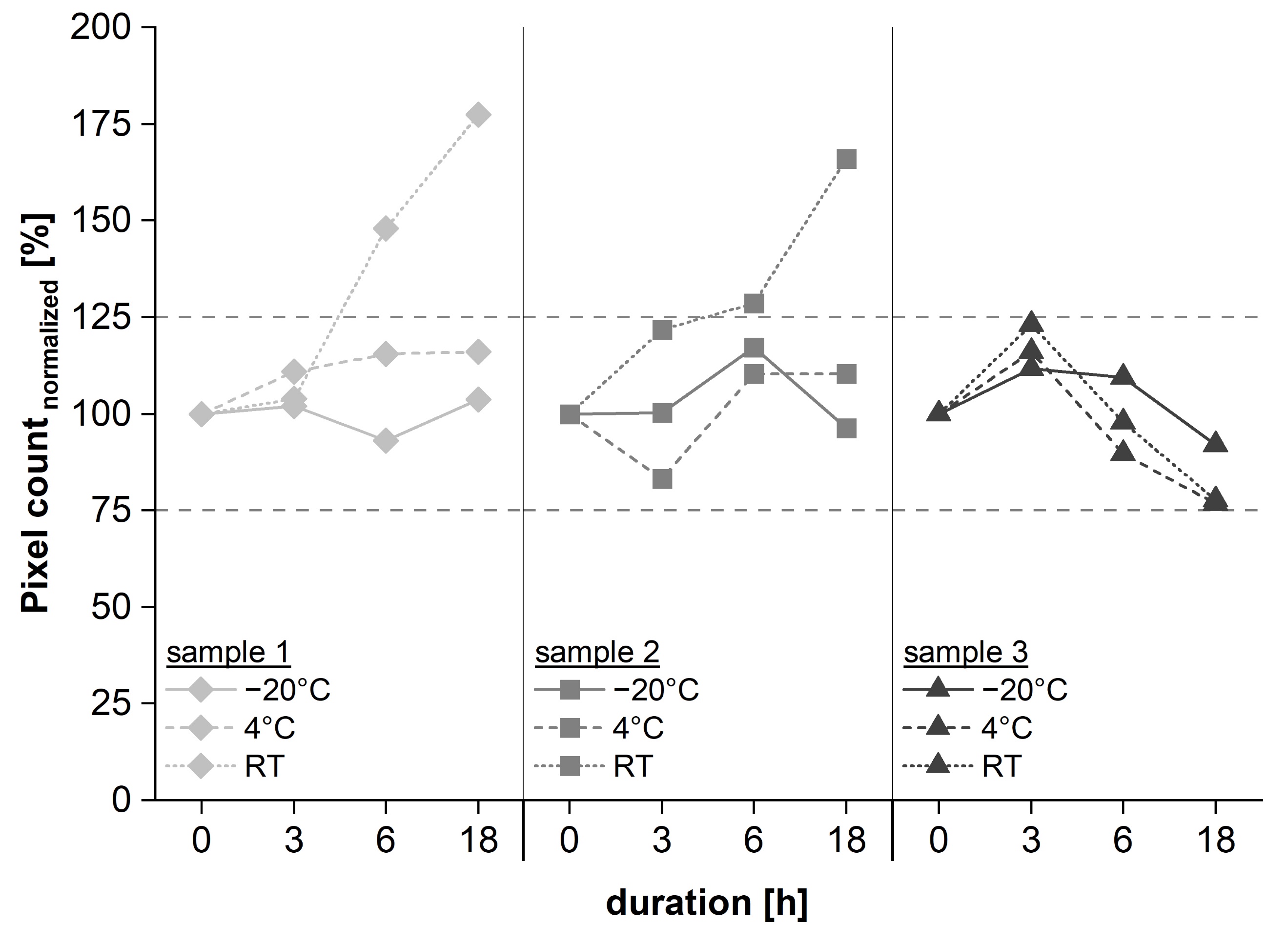


Figure S4. Evaluation of thermostability during transport or bench-top handling, related to Results.

To investigate thermostability, three crude fecal samples (sample 1 = diamonds, low readout; sample 2 = square, intermediate readout; sample 3 = triangle, high readout) were stored at −20 °C, 4 °C or 20 °C for 3, 6, or 18 h before they were frozen at −80 °C. Afterward, crude samples were homogenized, diluted 1:5 with sample buffer, and subjected to sFIDA analysis in quadruplicates. Normalized pixel counts were calculated using a non-stressed reference sample and plotted against storage time. Dashed lines indicate the tolerance range of ± 25%. Because none of the samples showed normalized pixel counts outside the tolerance, we assumed that sample stability at −20 °C and 4 °C is not affected within the analyzed time period of 3 to 18h. In contrast, simulated transport or bench-top handling at 20 °C exceeded the tolerance limit after 3 h (sample 1 and sample 2). A signal increase was observed for both samples, which can be explained by increasing autofluorescence caused by decay processes and bacterial growth at this temperature. Consequently, we have decided not to transport or handle the sample at RT and adapted the sFIDA procedure accordingly, i.e., by sufficiently cooling the fecal samples during the preanalytics.


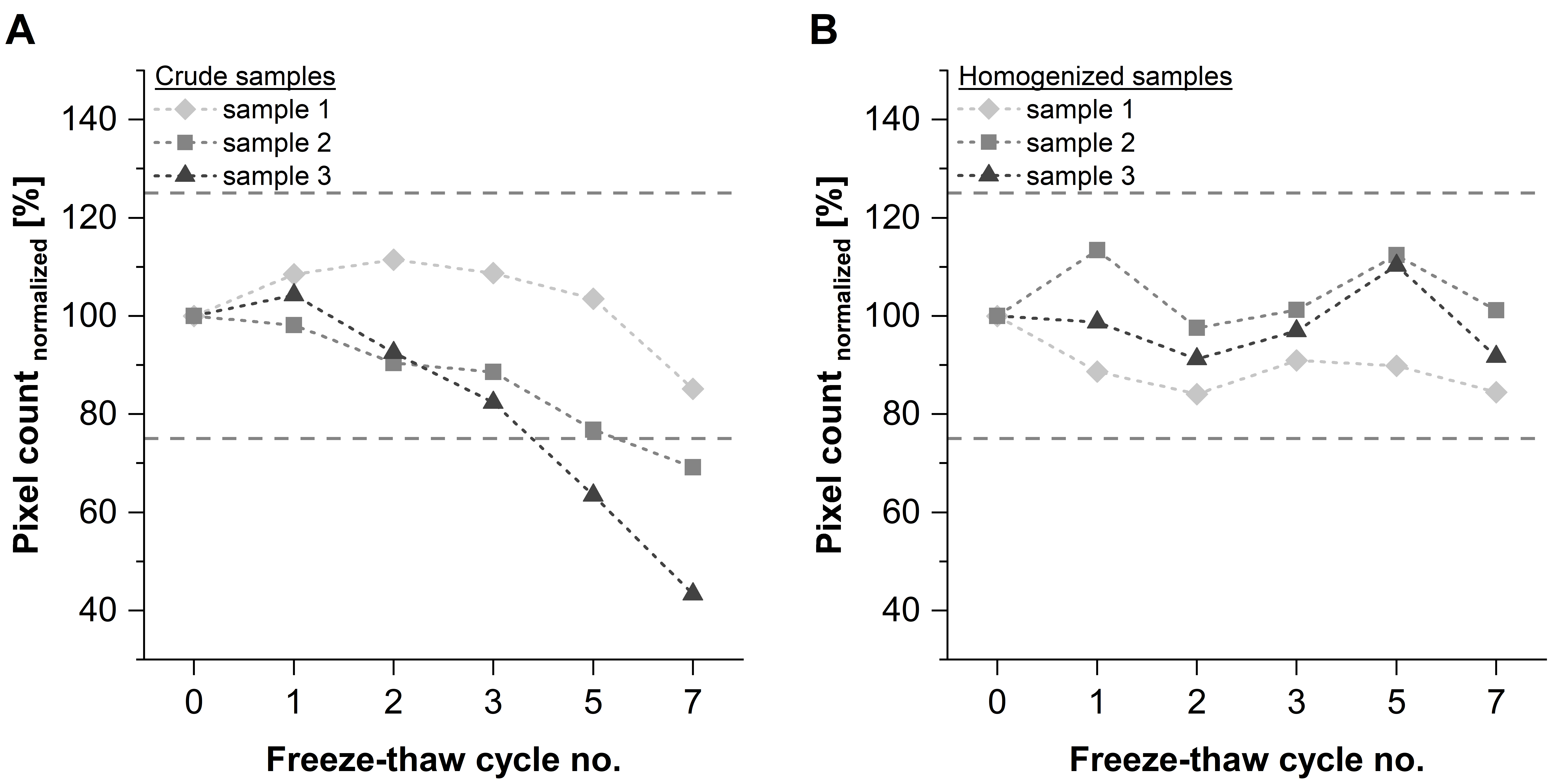


Figure S5. Influence of repeated freeze-thaw cycles on the stability of Aβ aggregates.

Normalized pixel counts of three crude (A) or homogenized (B) fecal samples (sample 1 = diamonds, low readout; sample 2 = square, intermediate readout; sample 3 = triangle, high readout) were plotted against freeze-thaw cycles. The tolerance range of ± 25% is depicted as dashed gray lines. In detail, for the crude control sample (sample 1, low readout), neither a signal reduction due to decreased sample stability nor a signal increase was observed. In contrast, a decreasing effect of the number of freeze-thaw cycles on the signal was determined for samples 2 and 3. In particular, repetitive freeze-thaw cycles dramatically reduced the stability of high-readout sample 3. Compared to the reference (no freeze-thaw cycle), a signal loss of 57% was recorded for this sample after thawing and freezing seven times. Considering crude sample 2, an out-of-tolerance value was also recorded at the seventh freeze-thaw cycle, with the measured signal dropping by 31% compared to the reference. In contrast to the crude fecal samples, no signal decrease was observed for the homogenized samples over all freeze-thaw cycles indicating high sample stability after homogenization.

**
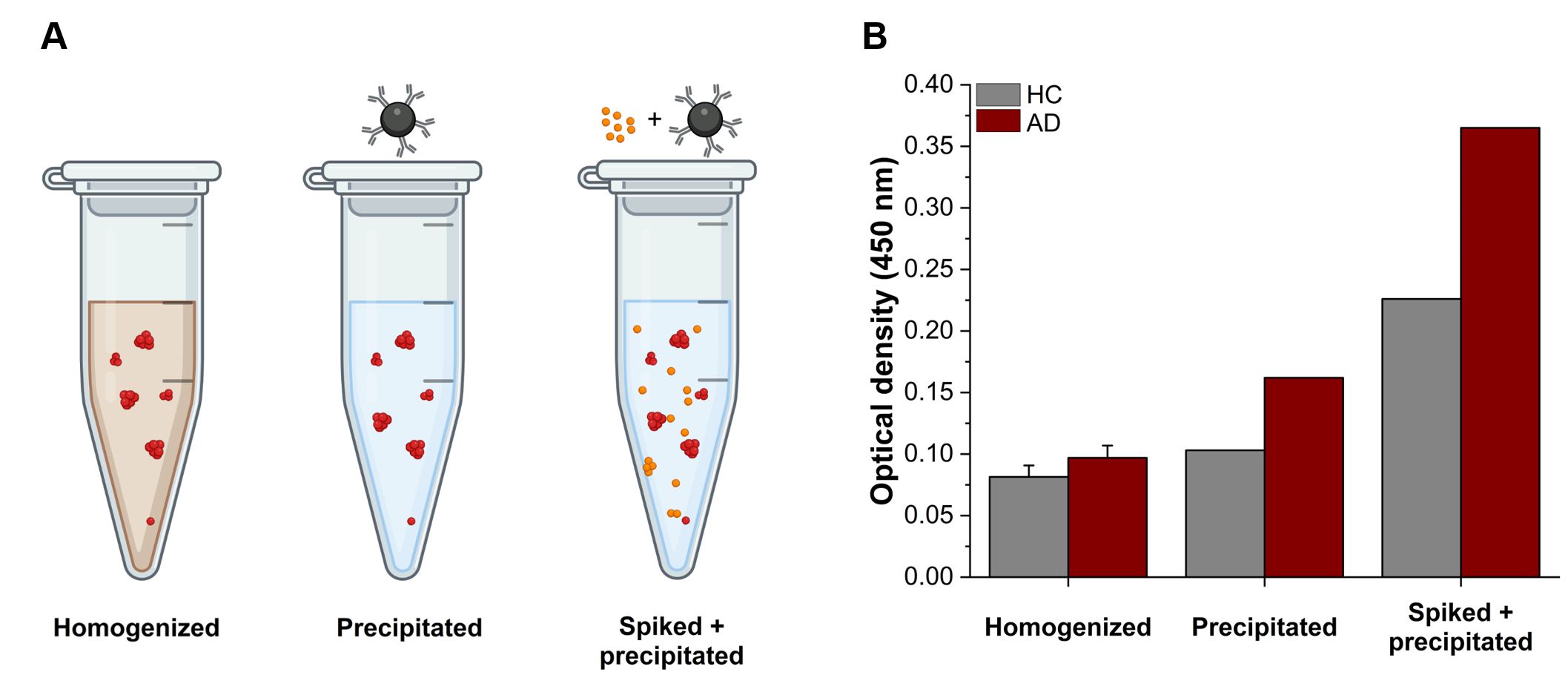
**

**Figure S6. Schematic illustration of sample treatment and confirming the presence of fecal Aβ species in those samples using ELISA, related to Results.**

To determine total Aβ (monomers and aggregates) in feces, two samples (gray: HC, red: AD patient) were analyzed using a human amyloid β (1−42) ELISA Assay kit (IBL International, Hamburg, Germany). While this ELISA was developed for plasma, CSF, and cell culture supernatants [13], it should at least be applicable for the qualitative detection of Aβ in the feces. We tested the effect of different sample treatments (“homogenized”, “precipitated”, and “spiked + precipitated”, schematic illustration in (**A**), *created with BioRender.com*.) on the discrimination between AD and HC. Signals (**B**) could be detected in those samples that were only homogenized, but no differentiation between both samples was possible. Sensitivity could only be increased by accumulation and elimination of the matrix by immunoprecipitation. This difference between control and AD could be further enhanced by adding Aβ1−42. NOTE. For the immunoprecipitation, a total of 1.5 g feces per donor were required to obtain 100 µL supernatant. Due to the limited amount of feces, only a single determination by ELISA was possible. Therefore, no standard deviation is shown. In case of homogenized samples, data are represented as mean with standard deviation.

# Supplementary Tables

Table S1. Overview of the clinical diagnosis of cognitively impaired study participants based on DemTect, Functional Activities Questionnaire (FAQ), Beck Depression Inventory II (BDI-II) or Memory Assessment Clinic-Questionnaire (MAC-Q) and assessment of functional abilities of daily living (ADL, preserved or impaired), related to Methods.

| **Sample**  **ID** | **Cohort** | **DemTect^b^** | **FAQ** | **BDI-II** | **MAC-Q** | **Functional ADL** | **Clinical diagnosis** |
| --- | --- | --- | --- | --- | --- | --- | --- |
| **1** | AD | 4 | NA | NA | NA | P | MCI |
| **2** | AD | NA | NA | NA | NA | I | dementia |
| **3** | AD | NA | NA | NA | NA | P | MCI |
| **4** | AD | 9 | NA | 4 | NA | P | MCI |
| **5** | AD | 10 | 0 | 2 | NA | P | MCI |
| **6** | AD | 9 | NA | 25 | NA | I | dementia |
| **7** | AD | 5 | NA | 30 | NA | P | MCI |
| **8** | AD | 12 | NA | 29 | NA | P | MCI |
| **9** | AD | 9 | NA | 29 | NA | P | MCI |
| **10** | AD | 9 | 5 | 11 | NA | P | MCI |
| **11** | AD | 2 | 2 | 3 | 10 | P | MCI |
| **12** | AD | 5 | 15 | 5 | 30 | I | dementia |
| **13** | AD | 4 | 0 | 6 | 24 | P | MCI |
| **14** | AD | 7 | 0 | 1 | 22 | P | MCI |
| **15** | AD | 12 | 2 | 7 | 26 | P | MCI |
| **16** | AD | 10 | 5 | 9 | 31 | P | MCI |
| **17** | AD | 5 | 26 | NA | 34 | I | dementia |
| **18** | AD | 10 | 6 | 9 | 27 | P | MCI |
| **19** | AD | 9 | 2 | 9 | 33 | P | MCI |
| **20** | AD | 12 | 0 | 14 | 25 | P | MCI |
| **21** | AD | 3 | 0 | 3 | 21 | P | MCI |
| **22** | AD | 11 | 4 | 6 | 24 | P | MCI |
| **23** | AD | 1 | 18 | 4 | 27 | P | MCI |
| **24** | AD | 12 | 5 | 8 | 26 | P | MCI |
| **25** | AD | 11 | 4 | 3 | 25 | P | MCI |
| **26** | AD | 18 | 0 | 14 | 29 | P | MCI |

^a^NOTE. Raw scores were transformed as described by Kalbe et al. [14].

^b^Abbreviations: AD, Alzheimer’s disease; BDI-II, Beck Depression Inventory II; F, female; FAQ, Functional Activities Questionnaire; I, impaired; M, male; MAC-Q, Memory Assessment Clinic-Questionnaire; MCI, mild cognitive impairment; NA, not applicable; P, preserved.

Table S2. Results of normal distribution tests for molar Aβ aggregate concentrations in fecal samples, related to Results.

| **Normal distribution test** | **All samples** | **AD samples** | **HC samples** |
| --- | --- | --- | --- |
| **Shapiro−Wilk** | 2.51*10^−14^ | 3.37*10^−8^ | 0.003 |
| **Lilliefors** | 3.99*10^−19^ | 9.71*10^−7^ | 0.109 |
| **Kolmogorov Smirnov** | 1.24*10^−6^ | 0.012 | 0.513 |
| **Anderson−Darling** | 7.11*10^−31^ | 6.43*10^−12^ | 0.018 |

^a^Abbreviations: AD, Alzheimer’s disease; HC, healthy controls

Table S3. The coefficient of variation, calculated calibration curve, coefficient of determination and the limit of detection of each experiment for Aβ-coated SiNaPs, IQC samples, and fecal samples, related to Results.

|  | **Experiment 1** | **Experiment 2** | **Mean** |
| --- | --- | --- | --- |
| **CV%** | S: 11.54 | S: 15.15 | S: 13.35 |
|  | I: 10.91 | I: 11.30 | I: 11.11 |
|  | F: 19.36 | F: 18.13 | F: 18.74 |
| **Calibration curve** | Range: 0.32 fM − 1026 fM | Range: 1.03 fM − 1026 fM | - |
|  | y = 1.536x + 6.984 | y = 1.118x + 10.88 | - |
|  | R^2^ = 0.9709 | R^2^ = 0.9886 | R^2^ = 0.9798 |
| **LoD [fM]** | 2.27 | 1.09 | 1.68 |

^a^NOTE. CV% are defined as the ratio of standard deviation and the mean of the respective sample quadruplicates. The calibration curve was calculated based on the pixel counts of Aβ-coated SiNaPs using linear regression (readouts were weighted with 1/readout).

^b^Abbreviations: CV%, coefficient of variation; S, Aβ-coated SiNaPs; I, IQC samples; F, fecal samples; R^2^, coefficients of determination; LoD, the limit of detection.

Table S4. Clinical information, Bristol scale scores, and mean Aβ aggregate concentrations with the standard deviation of the individual subjects, related to Results, Figure 5B and Figure 5C.

| Sample ID | Cohort | Cognition | Clinical symptoms | Sex | Age [years] | Bristol scale | Aβ40 [pg/mL] | Aβ42 [pg/mL] | Ratio Aβ40/Aβ42 | tTau [pg/mL] | pTau pg/mL] | Amyloid PET | Tau PET | Fecal Aβ aggregate  concentration ± SD [fM] |
| --- | --- | --- | --- | --- | --- | --- | --- | --- | --- | --- | --- | --- | --- | --- |
| 1 | AD | impaired | MCI | M | 75 | 5 | NA | 475 | NA | 280 | 46 | pos | pos | 1852.5 ± 174.7 |
| 2 | AD | impaired | dementia | F | 84 | 4 | NA | NA | NA | NA | NA | pos | pos | 3430.8 ± 773.7 |
| 3 | AD | impaired | MCI | M | 60 | 4 | 7224 | 550.4 | 0.076 | 426.9 | 59 | NA | NA | 50.9 ± 3.53 |
| 4 | AD | impaired | MCI | M | 68 | 5 | 5172 | 321.3 | 0.06 | 479.4 | 86 | NA | NA | 49 ± 6.31 |
| 5 | AD | impaired | MCI | M | 79 | 3 | NA | NA | NA | NA | NA | pos | NA | 49.4 ± 8.63 |
| 6 | AD | impaired | dementia | M | 76 | 3 | 8537 | 586.4 | 0.07 | 671.1 | 119 | NA | NA | 798.9 ± 364.6 |
| 7 | AD | impaired | MCI | M | 67 | 5 | 4905 | 248.5 | 0.051 | 468.4 | 69 | NA | NA | 322.4 ± 45.6 |
| 8 | AD | impaired | MCI | M | 48 | 5 | NA | 435 | NA | NA | 84 | NA | NA | 273.3 ± 33.9 |
| 9 | AD | impaired | MCI | F | 76 | 5 | 9592 | 851.6 | 0.089 | 364.7 | 68 | NA | NA | 40.1 ± 3.78 |
| 10 | AD | impaired | MCI | M | 69 | 3 | NA | NA | NA | NA | NA | pos | NA | 28.7 ± 3.97 |
| 11 | AD | impaired | MCI | F | 79 | 4 | 6890 | 515.1 | 0.075 | 583.7 | 99 | NA | NA | 139.7 ± 44.1 |
| 12 | AD | impaired | dementia | M | 79 | 4 | 7483 | 635.77 | 0.085 | 614.43 | 83 | NA | NA | 215.3 ± 26.2 |
| 13 | AD | impaired | MCI | F | 71 | 5 | 10362 | 621.89 | 0.06 | 561.73 | 110 | NA | NA | 462.9 ± 143.9 |
| 14 | AD | impaired | MCI | M | 72 | 3 | 12703 | 1138.1 | 0.09 | 602 | 100 | NA | NA | 120.6 ± 15.7 |
| 15 | AD | impaired | MCI | M | 69 | 5 | 4961 | 401.4 | 0.081 | 548 | 118 | NA | NA | 45.5 ± 6.66 |
| 16 | AD | impaired | MCI | F | 75 | 4 | 8941 | 806.8 | 0.09 | 799 | 139 | NA | NA | 53.6 ± 12.4 |
| 17 | AD | impaired | dementia | F | 67 | 4 | 8982 | 787.7 | 0.088 | 815 | 131 | NA | NA | 387.1 ± 46.2 |
| 18 | AD | impaired | MCI | F | 81 | 4 | 6935 | 566.4 | 0.082 | 674 | 97 | NA | NA | 268.7 ± 48.4 |
| 19 | AD | impaired | MCI | M | 65 | 4 | 6507 | 488.8 | 0.075 | 641 | 129 | NA | NA | 665.9 ± 312.8 |
| 20 | AD | impaired | MCI | F | 83 | 5 | 5272 | 487.4 | 0.092 | 458 | 65 | NA | NA | 65.6 ± 4.59 |
| 21 | AD | impaired | MCI | F | 67 | 3 | 9276 | 749.1 | 0.081 | 887 | 164 | NA | NA | 15.5 ± 1.66 |
| 22 | AD | impaired | MCI | F | 73 | 6 | 12451 | 806 | 0.065 | 753 | 98 | NA | NA | 165.2 ± 46.4 |
| 23 | AD | impaired | MCI | M | 81 | 6 | 7248 | 537 | 0.074 | 531 | 100 | NA | NA | 40.3 ± 4.51 |
| 24 | AD | impaired | MCI | M | 64 | 4 | 10503 | 850.2 | 0.081 | 699 | 106 | NA | NA | 111.5 ± 11.6 |
| 25 | AD | impaired | MCI | M | 60 | 5 | 11101 | 1003.6 | 0.09 | 640 | 126 | NA | NA | 62.6 ± 12.5 |
| 26 | AD | impaired | MCI | F | 60 | 6 | 12364 | 681.5 | 0.055 | 778 | 125 | NA | NA | 2.2 ± 0.24 |
| 27 | HC | normal | no | A | A | 5 | NA | NA | NA | NA | NA | NA | NA | 104 ± 25.7 |
| 28 | HC | normal | no | A | A | 6 | NA | NA | NA | NA | NA | NA | NA | 190.7 ± 53.2 |
| 29 | HC | normal | no | A | A | 5 | NA | NA | NA | NA | NA | NA | NA | 49.2 ± 8 |
| 30 | HC | normal | no | A | A | 5 | NA | NA | NA | NA | NA | NA | NA | 73.3 ± 6.7 |
| 31 | HC | normal | no | A | A | 5 | NA | NA | NA | NA | NA | NA | NA | 73.6 ± 7.9 |
| 32 | HC | normal | no | A | A | 5 | NA | NA | NA | NA | NA | NA | NA | 72.9 ± 22.2 |
| 33 | HC | normal | no | A | A | 4 | NA | NA | NA | NA | NA | NA | NA | 27.4 ± 3.9 |
| 34 | HC | normal | no | A | A | 4 | NA | NA | NA | NA | NA | NA | NA | 114.9 ± 27.3 |
| 35 | HC | normal | no | A | A | 3 | NA | NA | NA | NA | NA | NA | NA | 46.2 ± 14.5 |
| 36 | HC | normal | no | A | A | 5 | NA | NA | NA | NA | NA | NA | NA | 45.7 ± 9 |
| 37 | HC | normal | no | A | A | 5 | NA | NA | NA | NA | NA | NA | NA | 89.6 ± 15.4 |
| 38 | HC | normal | no | A | A | 5 | NA | NA | NA | NA | NA | NA | NA | 64 ± 12.4 |
| 39 | HC | normal | no | A | A | 6 | NA | NA | NA | NA | NA | NA | NA | 45.4 ± 5.7 |
| 40 | HC | normal | no | A | A | 6 | NA | NA | NA | NA | NA | NA | NA | 86.7 ± 15 |
| 41 | HC | normal | no | A | A | 6 | NA | NA | NA | NA | NA | NA | NA | 62.7 ± 12.1 |
| 42 | HC | normal | no | A | A | 5 | NA | NA | NA | NA | NA | NA | NA | 22.6 ± 4.4 |
| 43 | HC | normal | no | A | A | 6 | NA | NA | NA | NA | NA | NA | NA | 23.9 ± 7.1 |
| 44 | HC | normal | no | A | A | 6 | NA | NA | NA | NA | NA | NA | NA | 56.6 ± 12.7 |
| 45 | HC | normal | no | A | A | 5 | NA | NA | NA | NA | NA | NA | NA | 58 ± 14.7 |
| 46 | HC | normal | no | A | A | 6 | NA | NA | NA | NA | NA | NA | NA | 198.5 ± 49.6 |
| 47 | HC | normal | no | A | A | 4 | NA | NA | NA | NA | NA | NA | NA | 67.1 ± 7.4 |
| 48 | HC | normal | no | A | A | 6 | NA | NA | NA | NA | NA | NA | NA | 32.9 ± 5.8 |
| 49 | HC | normal | no | A | A | 5 | NA | NA | NA | NA | NA | NA | NA | 99.3 ± 20.2 |
| 50 | HC | normal | no | A | A | 4 | NA | NA | NA | NA | NA | NA | NA | 11.3 ± 3.2 |
| 51 | HC | normal | no | A | A | 5 | NA | NA | NA | NA | NA | NA | NA | 5.1 ± 1.2 |
| 52 | HC | normal | no | A | A | 5 | NA | NA | NA | NA | NA | NA | NA | 96.4 ± 16.6 |
| 53 | HC | normal | no | A | A | 6 | NA | NA | NA | NA | NA | NA | NA | 15.6 ± 2 |
| 54 | HC | normal | no | A | A | 6 | NA | NA | NA | NA | NA | NA | NA | 33.2 ± 4.6 |
| 55 | HC | normal | no | A | A | 6 | NA | NA | NA | NA | NA | NA | NA | 36.3 ± 6.5 |
| 56 | HC | normal | no | A | A | 5 | NA | NA | NA | NA | NA | NA | NA | 13.6 ± 1.6 |
| 57 | HC | normal | no | A | A | 4 | NA | NA | NA | NA | NA | NA | NA | 1.3 ± 0.5 |

^a^Abbreviations: A, anonymized, Aβ, amyloid-beta, AD, Alzheimer’s disease; MCI, mild cognitive impairment; HC, healthy controls; NA, not applicable, PET, Positron emission tomography, SD, standard deviation.

Table S5. Spearman coefficient of correlation values for the analyses between fecal Aβ aggregate levels and seven fecal biomarkers indicating gut inflammation and increased permeability of intestinal membranes (bile acid, lipids, calprotectin, IgA, α-1-antitrypsin, hemoglobin, and albumin), related to Results.

| **Biomarker** | **Bile acid** | **Lipids** | **Calpro-tectin** | **IgA** | **α1-Anti-trypsin** | **Hemo-globin** | **Albumin** | **Aβ aggregates** |
| --- | --- | --- | --- | --- | --- | --- | --- | --- |
| **Bile acid** | 1 | 0.5181* | −0.1580 | −0.1596 | −0.0164 | 0.2236 | 0.0039 | −0.1108 |
| **Lipids** |  | 1 | −0.2998 | 0.0778 | −0.2737 | 0.0854 | 0.1134 | −0.3805 |
| **Calprotectin** |  |  | 1 | 0.5941* | 0.5845* | 0.4669 | 0.5911* | 0.1489 |
| **IgA** |  |  |  | 1 | 0.2336 | 0.2423 | 0.6071* | −0.0118 |
| **α1-Antitrypsin** |  |  |  |  | 1 | 0.3872 | 0.0731 | 0.3564 |
| **Hemoglobin** |  |  |  |  |  | 1 | 0.5965* | −0.0121 |
| **Albumin** |  |  |  |  |  |  | 1 | −0.3665 |
| **Aβ aggregates** |  |  |  |  |  |  |  | 1 |

^a^NOTE. Significant correlations are indicated by * *p*: ≤ 0.05.

**Table S6.** *p*-**values of two-sided Mann−Whitney U test for pairwise comparisons of measured fecal biomarker concentrations,** related to Results**.**

| Biomarker | AD (n = 7) | HC (n = 8) | *p*-value |
| --- | --- | --- | --- |
| Bile acid [mmol/100 g] | 350 ± 91.7 | 272 ± 70.5 | 0.1321 |
| Lipids wt% | 1.6 ± 0.7 | 1.6 ± 0.6 | 0.8608 |
| Calprotectin [µg/g] | 40.3 ± 27.5 | 53.5 ± 32.5 | 0.3524 |
| IgA [µg/g] | 1444 ± 2041 | 1716 ± 1433 | 0.2699 |
| α1-Antitrypsin [µg/g] | 39.1 ± 22.7 | 61.8 ± 47.6 | 0.1753 |
| Hemoglobin [µg/g] | 0.3 ± 0 | 0.4 ± 0.3 | 0.2036 |
| Albumin [µg/g] | 4.1 ± 2.0 | 9.8 ± 10.9 | 0.1870 |

^a^NOTE. No significant differences of fecal biomarker concentrations between AD patients and HC were found. ^b^Abbreviations: AD, Alzheimer’s disease; HC, healthy controls.

# Supplemental References

[1] Kass, B., et al., *Aβ oligomer concentration in mouse and human brain and its drug-induced reduction ex vivo.* Cell Rep Med, 2022. **3**(5): p. 100630.

[2] Vagenende, V., M.G. Yap, and B.L. Trout, *Mechanisms of protein stabilization and prevention of protein aggregation by glycerol.* Biochemistry, 2009. **48**(46): p. 11084-96.

[3] Rose, C., et al., *The Characterization of Feces and Urine: A Review of the Literature to Inform Advanced Treatment Technology.* Crit Rev Environ Sci Technol, 2015. **45**(17): p. 1827-1879.

[4] Stephen, A.M. and J.H. Cummings, *The microbial contribution to human faecal mass.* J Med Microbiol, 1980. **13**(1): p. 45-56.

[5] Phadia-AB. *Stool Extraction Kit plus / Stool Extraction Buffer plus, instruction for use: 200/250/2500/5000-6665-020 / UK, version 2021-09-14.* . 2021; Available on <https://dfu.phadia.com/Data/Pdf/6130cb9570eec660e274806b.pdf> [Accessed 23 January 2023].].

[6] ORGENTEC-Diagnostika-GmbH. *Calprotectin, instruction for use: ORG 580_4, version ORG 580_IFU_EN_QM140866_2022-03-08_4.* 2022; Available on <https://products.orgentec.com/pdfs/ifu/ORG%20580_IFU_EN_QM140866_2022-03-08_4.pdf> [Accessed 23 January 2023].].

[7] Bühlmann-Laboratories. *Bühlmann Smart-Prep - Faecal sample preparation kit, Instruction for use: B-CAL-RD, version 2010-11-10.* 2010; Available on <https://www.buhlmannlabs.ch/wp-content/uploads/2015/01/B-CAL-RD_101110.pdf> [Accessed 23 January 2023].].

[8] Immundiagnostik-AG. *IDK Hemoglobin ELISA: For the in vitro determination of hemoglobin in stool, instruction for use: K 7816D, K7816D.20, version 2022-02-22.* 2022; Available on <https://www.immundiagnostik.com/media/pages/testkits/k-7816d/9a63870b70-1663639307/k7816d_2022-02-22_haemoglobin.pdf> [Accessed 23 January 2023].].

[9] Demeditec-Diagnostics-GmbH. *Calprotectin ELISA, instruction for use: DE849 V211014/DLB, version 2021-11-25.* . 2021; Available on <https://www.demeditec.de/de/produkte/calprotectin-elisa-de849/ifu-de849-calprotectin-elisa-211125-e.pdf> [Accessed 23 January 2023].].

[10] Andreasson, U., et al., *A Practical Guide to Immunoassay Method Validation.* Front Neurol, 2015. **6**: p. 179.

[11] Blömeke, L., et al., *Quantitative detection of α-Synuclein and Tau oligomers and other aggregates by digital single particle counting.* NPJ Parkinsons Dis, 2022. **8**(1): p. 68.

[12] Park, J.Y. and L.J. Kricka, *Chapter 5.3 - Interferences in Immunoassay*, in *The Immunoassay Handbook (Fourth Edition)*, D. Wild, Editor. 2013, Elsevier: Oxford. p. 403-416.

[13] IBL-international. *Amyloid-beta (1-42) (FL) ELISA Instruction for use Code No. 27719, version 2.* 2016; Available on <https://www.ibl-america.com/content/elisa/27719.pdf> [Accessed 23 January 2023].].

[14] Kalbe, E., et al., *DemTect: a new, sensitive cognitive screening test to support the diagnosis of mild cognitive impairment and early dementia.* Int J Geriatr Psychiatry, 2004. **19**(2): p. 136-43.
